# pY RNA1-s2: A Highly Retina-Enriched Small RNA That Selectively Binds to Matrin 3 (Matr3)

**DOI:** 10.1371/journal.pone.0088217

**Published:** 2014-02-18

**Authors:** Fumiyoshi Yamazaki, Hyun Hee Kim, Pierre Lau, Christopher K. Hwang, P. Michael Iuvone, David Klein, Samuel J. H. Clokie

**Affiliations:** 1 Section on Neuroendocrinology, Program in Developmental Endocrinology and Genetics, The *Eunice Shriver Kennedy* National Institute of Child Health and Human Development, National Institutes of Health, Bethesda, Maryland, United States of America; 2 Division of intramural research, National Institute of Neurological Disorders and Stroke, National Institutes of Health, Bethesda, Maryland, United States of America; 3 Departments of Ophthalmology and Pharmacology, Emory University School of Medicine, Atlanta, Georgia, United States of America; Colorado State University, United States of America

## Abstract

The purpose of this study was to expand our knowledge of small RNAs, which are known to function within protein complexes to modulate the transcriptional output of the cell. Here we describe two previously unrecognized, small RNAs, termed pY RNA1-s1 and pY RNA1-s2 (processed Y RNA1-stem −1 and −2), thereby expanding the list of known small RNAs. pY RNA1-s1 and pY RNA1-s2 were discovered by RNA sequencing and found to be 20-fold more abundant in the retina than in 14 other rat tissues. Retinal expression of pY RNAs is highly conserved, including expression in the human retina, and occurs in all retinal cell layers. Mass spectrometric analysis of pY RNA1-S2 binding proteins in retina indicates that pY RNA1-s2 selectively binds the nuclear matrix protein Matrin 3 (Matr3) and to a lesser degree to hnrpul1 (heterogeneous nuclear ribonucleoprotein U-like protein). In contrast, pY RNA1-s1 does not bind these proteins. Accordingly, the molecular mechanism of action of pY RNA1-s2 is likely be through an action involving Matr3; this 95 kDa protein has two RNA recognition motifs (RRMs) and is implicated in transcription and RNA-editing. The high affinity binding of pY RNA1-s2 to Matr3 is strongly dependent on the sequence of the RNA and both RRMs of Matr3. Related studies also indicate that elements outside of the RRM region contribute to binding specificity and that phosphorylation enhances pY RNA-s2/Matr3 binding. These observations are of significance because they reveal that a previously unrecognized small RNA, pY RNA1-s2, binds selectively to Matr3. Hypothetically, pY RNA1-S2 might act to modulate cellular function through this molecular mechanism. The retinal enrichment of pY RNA1-s2 provides reason to suspect that the pY RNA1-s2/Matr3 interaction could play a role in vision.

## Introduction

Small RNAs have come to the forefront of research on non-coding RNAs and growing evidence indicates that they play critical and diverse roles in the regulation of cellular function. Small RNAs can be divided into several classes. Perhaps the best studied is the microRNAs (miRNA), which function to reduce or modulate protein levels by mRNA destabilization or translational inhibition [1]. This is caused by the complementary base pairing of miRNAs and cognate mRNA within a protein complex known as RISC (RNA induced silencing complex). Single stranded RNA is bound by argonaute 1 and 2 (Ago1 and 2), forming the core of the RISC complex. Further biochemical studies in HEK cells have revealed a multi-protein complex consisting of the RNase III endonuclease, DICER, TNRC6B (a poorly characterized, RRM containing protein), a putative RNA helicase MOV10, PRMT5 (an arginine methyl transferase) and the translation factor eEF1α [2].

In addition to miRNAs, there are several other classes of small RNAs that include piRNA (Piwi-associated RNA), which range in size from 24 to 30 nt. piRNAs were first identified in Drosophila from a repetitious region encompassing the beta subunit of the casein kinase II gene and originally described as repeat-associated small interfering RNA (rasiRNA) [3]. In contrast to miRNAs, piRNAs are not generated through the formation of dsRNA precursors and do not require the RNAse III enzymes for biogenesis (reviewed in [4]). piRNAs are generated from multiple loci and they are thought to silence transposon activity in trans, by binding to the protein PIWI in Drosophila (MILI in mammals) [5]. Loss-of-function studies indicate piRNAs are essential for germ cell development in spermatocytes as [6]. Further study of the silencing complex remains an area of intense study. Another class of small RNAs arise from condition- and tissue-specific processing of known small RNAs such as snoRNAs [7], scRNAs and tRNAs [8].

A relatively poorly understood group of small RNAs are Y RNAs, small 76 to 112 base RNA molecules with U6 promoters that are transcribed by RNA polymerase III [9]. They contain a poly U tail and bind with high affinity to the Ro and La proteins, thereby protecting the bound Y RNA from degradation [10]. There are five Y RNAs, designated Y RNA1, Y RNA2, Y RNA3, Y RNA4 and Y RNA5, that are reported to be ubiquitously expressed and also detectable in a range of model cell types including HeLa, HEp-2, MCF7, HF-V32, Jurkat and K-562 [11]. Y RNA isoforms have also been identified in a wide range of organisms ranging from the eubacterium Deinococcus radiodurans [12], c. elegans [13] to mammalian cells [11]. Phylogenetic analysis shows Y RNA are present in at least 27 genomes [14] and occur in syntenic regions [14]. Four Y RNA genes exist in the human genome with two in the mouse and rat genomes. Interestingly over a thousand Y RNA pseudogenes exist in the human genome whereas 48 exist in the rodent genome [14]. The first potential function ascribed to Y RNA is the requirement for chromosomal DNA replication; a discovery made some time after their initial discovery in 1980. Loss of function experiments using siRNA against two regions of the Y RNA showed a reduced ability of HeLa cells to replicate [15]. Intriguingly, processed Y RNA 5 and 3 (in human cell lines) have been shown to occur independently of miRNA biogenesis and functional pathways [16]. Until this current report, binding partners of processed Y RNA have not been identified.

Studies on small RNAs in the pineal gland, using RNA Sequencing have profiled the population of miRNAs, which includes over 400 miRNAs [17]. Of special interest is the finding that the miRNA population profile of the pineal gland is very similar to that of the retina, which is thought to share a common evolutionary origin [18]. Functional studies have indicated that one of these miRNAs regulates the developmental expression of a key enzyme in melatonin synthesis, arylalkylamine N-acetyltransferase. Subsequent analysis of the RNA Sequencing data from that study [18] provided the first evidence of the existence of two processed Y RNA1s (pY RNA1s).

The objective of this investigation was to extend studies of pY RNA1s by characterizing the tissue distribution pattern and identifying binding partners of pY RNA1-s2. This aim was achieved through the discoveries of exceptionally high abundance in the vertebrate retina and of very strong and selective affinity for a nuclear matrix protein.

## Results

### Identification of processed Y RNA (pY RNA)

**Figure 1:**
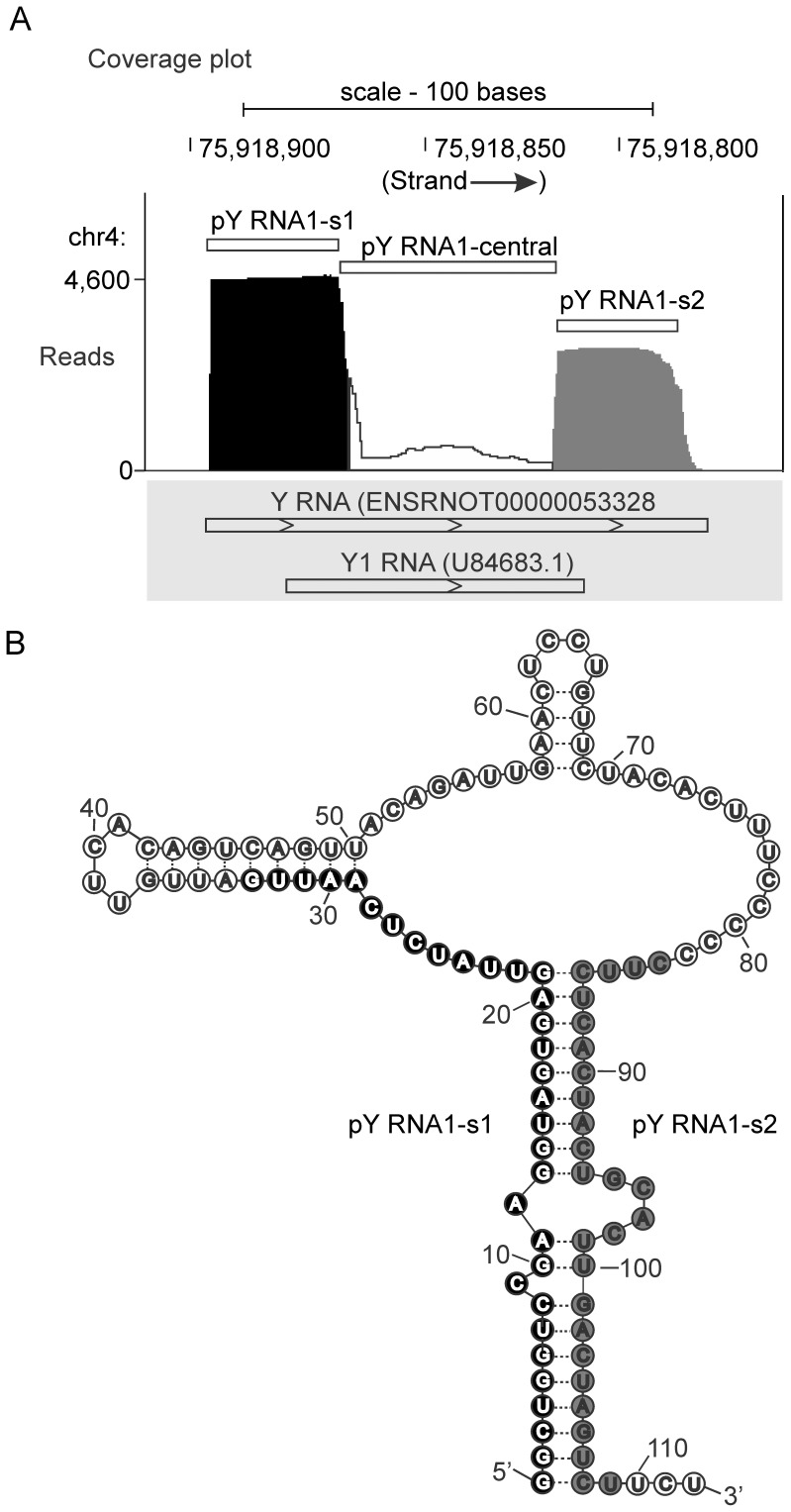
Massively parallel sequencing of small RNAs purified from the rodent pineal gland [17]. **A.** Coverage plot indicating the number of reads aligned to the rat genome (build rn4) using the Novoalign alignment tool and visualized using the UCSC genome browser [49]. The aligner was used with ‘miRNA mode’ switched on and multiple good–quality alignments (of each read) were reported, full details in “Materials and Methods”. Reads that align to the small cytoplasmic Y RNA1 and are distributed into two distinct populations, indicated by black (5′ stem region; pY RNA1-s1) and grey (3′ stem region; pY RNA1-s2) shading. Approximately 4,600 reads map to Y RNA-s1 and ∼3,000 to pY RNA1-s2. The transcripts indicated in the grey box are the Ensembl prediction for Y RNA1 and the incomplete Genbank entry for Y RNA1 in rat (U84683.1). Approximately 20 bases are missing from the 5′ and 3′ end GenBank entry U84683.1, as indicated by the histogram above. **B.** Secondary structure of the Y RNA gene adapted from [22], modified to reflect the locations of pY RNA1-S1 and pY RNA1-s2: the location of processed small RNAs is indicated with black and grey circles (pY RNA1-s1 and pY RNA1-s2, respectively) that correspond to the coverage plot in **A**.

Data mining of small RNA data obtained from the pineal gland [17] revealed that ∼8,000 reads, out of a total library size of ∼6 million reads, align to the small cytoplasmic RNA: Y RNA1 (Figure 1); existence of Y RNA1 is predicted using sequences from RFAM [19] and miRBase [20] (prediction performed by Ensembl [21]). The majority of reads map to the 5′ and 3′ ends of Y RNA1 (Figure 1A), with the remainder mapping contiguously within the central region (Figure 1A, Figure S1A in File S1). The secondary structure of Y RNA was reported in [22] and is re-drawn to indicate the most abundant Y RNA-derived sequences (pY RNA1-s1 and pY RNA1-s2) comprise the main stem of the YRNA structure (Figure 1B). The size of the pY RNA1-s1/s2 fragments is consistent with the size of other processed Y RNA homologs: Y RNA3 and Y RNA5 [23].

Mapping quality was assessed by examining reads that align to the three regions encompassing Y RNA1 (Figure 1A); the number of times a read mapped to each region within Y RNA1 and other genomic locations is reported in Table 1. This analysis indicates that reads aligning to pY RNA1-s2 are 98% uniquely mapped (Table 1), versus 84% for pY RNA1-s1; based on this, detailed investigations of pY RNA1-s2 were initiated.

**Table 1:** Distribution of pineal RNA sequencing reads mapping for Y RNA regions.

Y RNA region	Total # of reads	Number of locations			Percent of reads at location			Total (%)
		1	2 to 5	6 to 10	1	2 to 5	6 to 10	
pY RNA-s1	3,787	3,173	469	145	84	12	4	103
Central region	1,196	976	313	8	82	18	0.01	214
pY RNA-s2	2,796	2,720	66		98	2		120

Short reads aligning to the Y RNA region are divided into three groups. “Total # of reads at location” indicates how many reads were aligned to each Y RNA region. “Number of locations” provides a breakdown of the total numbers – reporting genome-wide multiple mapping characteristics: of the three bins; “1” - uniquely mapped, “2 to 5” – mapping 2,3,4 or 5 times and “6 to 10” – mapping 6,7,8,9 or 10 times to the rat genome. These same figures are calculated as a percentage – “Percent of reads at location” - and show that pY RNA1-s2 contains 98% uniquely mapped reads.

A BLAST search using the 27 nt sequence of pY RNA1-s2 identified two partial matches. One partial match contains 10 mismatches to a homologous Y RNA1, located on a different chromosome (GenBank entry: X69719) [11] (Figure S1B in File S1). The other match is unique to the GenBank entry: U84683.1 (unpublished, [24]). This entry has 100% identity with 74 bases of the central region of the predicted Ensembl Y RNA1, but lacks the 5′ and 3′; ends of the sequenced Y RNA1 presented herein (Figure 1A, grey panel). These data show complete coverage across the Ensembl predicted Y RNA1 (Figure 1A). The contiguously aligned small reads therefore represent the first reported cloning of Y RNA1 from rat, which has mouse (mY1 RNA) and human (RNY1) homologs [9]. Full length rat Y RNA1 was confirmed by conventional Sanger sequencing of cDNA derived from pineal tissue (data not shown).

### pY RNA1-s2 is highly enriched and present throughout multiple layers of the retina

**Figure 2:**
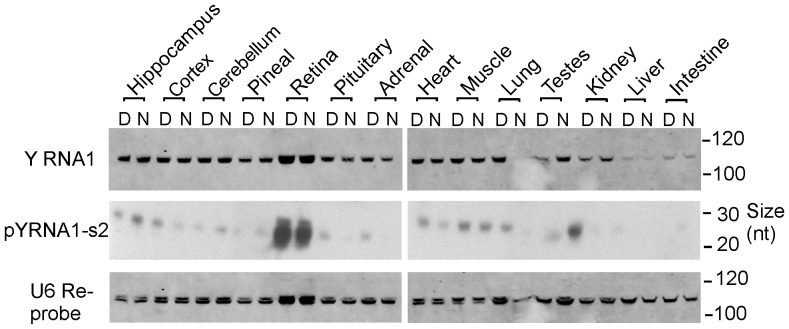
Tissue specific expression of pY RNA1-s2. Northern blotting of RNA extracted from the indicated tissue obtained during the Day (ZT 7) and Night (ZT 19). Tissues from three rats were pooled and RNA was extracted as described in “Materials and Methods”. Equal amounts (1 µg) of total RNA were separated on 15% TBE-Urea polyacrylamide gels, transferred and chemically cross-linked to the membrane, followed by probing with an LNA™-probe, designed against pY RNA1-s2. For further details see “Materials and Methods”. The upper panel; full-length Y RNA; middle panel, pY RNA1-s2; lower panel, U6 as loading control. This analysis was repeated two more times with similar results.

The relative abundance of pY RNA1-s2 was determined using Northern blot analysis of fourteen neuronal and peripheral rat tissues (Figure 2). The LNA probe used for this purpose specifically recognizes the sequence of pY RNA1-s2 and the full length Y RNA1 parent. The abundance of full length Y RNA1 was relatively similar among the 14 tissues examined, in contrast to the processed form: pY RNA1-s2, which was >20 times more abundant in retina (Figure 2). Significant night/day difference in the abundance of pY RNA1-s2 abundance was not observed.

**Figure 3:**
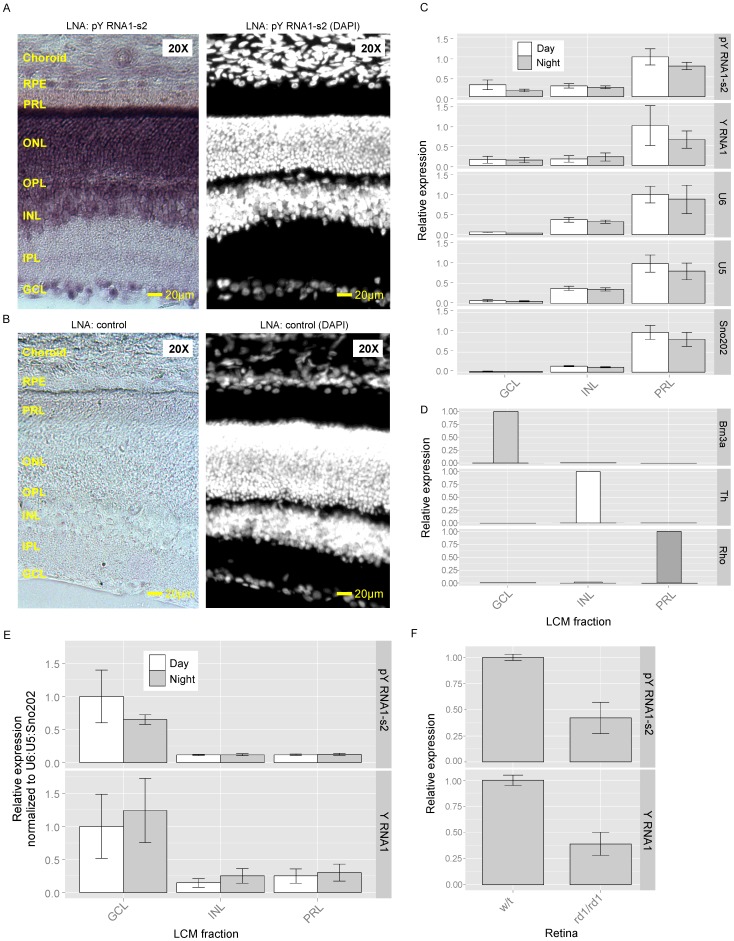
A. pY RNA1-s2/Y RNA1 is enriched in the ganglion cell layer (GCL) and photoreceptive layers (PRL) of retina. An antisense dioxygenin-labelled Locked Nucleic Acid (LNA):DNA probe was used to identify the location of Y RNA1 and pY RNA1-s2 in the retina of rat; full details are given in “Materials and Methods”. Intense staining is evident in the GCL and PRL, in particular the region corresponding to rods & cones. Other layers indicated are: IPL, inner plexiform layer; INL, inner nuclear layer; OPL, outer plexiform layer; ONL, outer nuclear layer; Rods & Cones; RPE, Retinal Pigmented Epithelium. Similar results were obtained with tissue from two other individuals. **B.** DAPI staining of retina cross section. The retinal section was DAPI stained to aid identification of cell morphology. **C.** Quantitative real-time PCR of small RNA species in regions of a mouse retina separated by laser capture micro-dissection (LCM). RNA was extracted from the indicated sections representing the GCL, INL and PRL and analyzed for the presence of pY RNA1-s2, Y RNA (full length Y RNA) and three control RNAs: U6, U5 and sno202. Retina were collected and dissected from animals housed in a 12∶12 L∶D6 lighting (day, white bars; and grey, night bars). Expression is reported as relative to the maximum signal for each small RNA. cDNA libraries were prepared using the QuanitMiR system and the same cDNA library was used for each qRT-PCR analysis. Samples of RNA from each of four pools of tissue from three animals were analyzed; error bars represent SEM. **D.** Validation of laser capture microdissection (LCM). The results of LCM are validated by determining the abundance of three retinal layer-specific markers in the microdissected samples. The markers used are Brn3a (GCL), mGluR6 (rod and ON-type cone bipolar cells in INL), and Rho (rods in the PRL). Brn3a was only detected in the GCL sample and mGluR6 only in the INL sample; Rho was 500-fold more abundant in the PR sample as compared to the INL sample and was not detected in the GCL sample. **E.** Values of Y RNA1 and pY RNA1-s2 are reported relative to the maximum signal and normalized using the geometric mean of U6, U5 and Sno202. **F.** Relative quantitation of pY RNA1-s2 and Y RNA1 in a mouse model of retinal degeneration. Wild type retina: w/t, degenerated retina: rd1/rd1. Error bars are SEM, n = 3.

*In situ* hybridization was performed on retina obtained from rat and probed with a DIG-labeled LNA oligonucleotide antisense to pY RNA1-s2. Due to sequence similarity, this probe cannot distinguish between Y RNA1 and pY RNA1-S2; therefore, the in situ hybridization signals in Figure 3A represent the presence of Y RNA1 and/or pY RNA1-s2. This analysis reveals intense staining in the ganglion cell layer (GCL) and the photoreceptor cell layer (PRL). DAPI staining confirmed the identification of the retinal cellular layers. We performed *In situ hybridization* on fresh frozen liver sections to investigate binding specificity of the LNA probe (Figure S2A in File S1). Liver sections were examined based on the observation that pY RNA1-s2 is below the limit of detection in liver tissue, using Northern blotting (Figure 2), in contrast to the strong signal in the retina. This study was extended by examining rat retina using laser capture microdissection (LCM) to isolate the major cell layers of the retina: the photoreceptor layer (PRL), the inner nuclear layer (INL) and the ganglion cell layer (GCL) (Figure 3C). The purity of the dissection was confirmed using marker transcripts, each of which was found to be selectively expressed in a single layer (Figure 3D). pY RNA1-s2 was identified in all layers, although it was clearly enriched in the GCL and PRL layers. The small RNAs: U5, U6 and Sno202 were quantitated to assess the relative number of cells collected by LCM from each retina layer (Figure 3C). Interestingly the proportion of pY RNA1-s2 in relation to control RNA is greater in the GCL than the PRL (Figure 3E). To further confirm the presence of pY RNA1-s2 throughout the retina, we compared the expression in retinas from one year old wild type and C57BL/6J-*Pde6b^rd1-2J^*/J (rd1/rd1) mice, in which virtually all photoreceptors have degenerated. Consistent with the *in situ* hybridization and LCM-qRT-PCR data from rat retinas, the relative expression of pY RNA1-s2 was ∼60% reduced compared to wild type controls. Notably, this corresponds to the fraction of the total retinal cell population that are photoreceptors [18] (Figure 3F).

**Figure 4:**
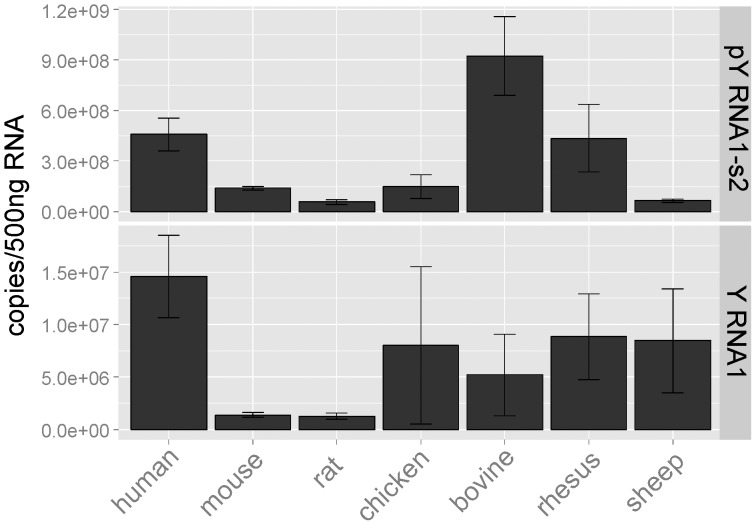
Presence of pY RNA1-s2 in mammalian and bird retinas. RNA was extracted from three retinas obtained from each of the indicated species; all samples were obtained during the day. Equal amounts of tissue were extracted using miRVana columns; cDNA libraries were created using the QuanitMiR system. The values are reported as copies of pY RNA1-s2/500 ng total RNA. It is apparent that the abundances of pY RNA1-s2 and of YRNA1 vary from species to species and that pY RNA1-s2 appears to be 50 to 100 times more abundant that Y RNA1, in all cases except the ovine retina. The Y RNA1 versus pY RNA1-s2 differences may reflect secondary structure differences which influence the efficiency of the RT reaction, resulting in greater amplification of pY RNA1-s2 over Y RNA1. Error bars are SEM.

The presence of pY RNA1-s2 was explored in retinas of other vertebrates, using quantitative real-time PCR, including mouse, chicken, cow, sheep, monkey and human. This revealed pY RNA1-s2 was present in retinas of all species tested (Figure 4, upper panel) at generally similar levels, with the lowest levels recorded in rodents (mouse and rat). Y RNA1 was also quantitated and shows a similar pattern (Figure 4, lower panel).

### pY RNA1-s2 exists in cytoplasm and nucleus

**Figure 5:**
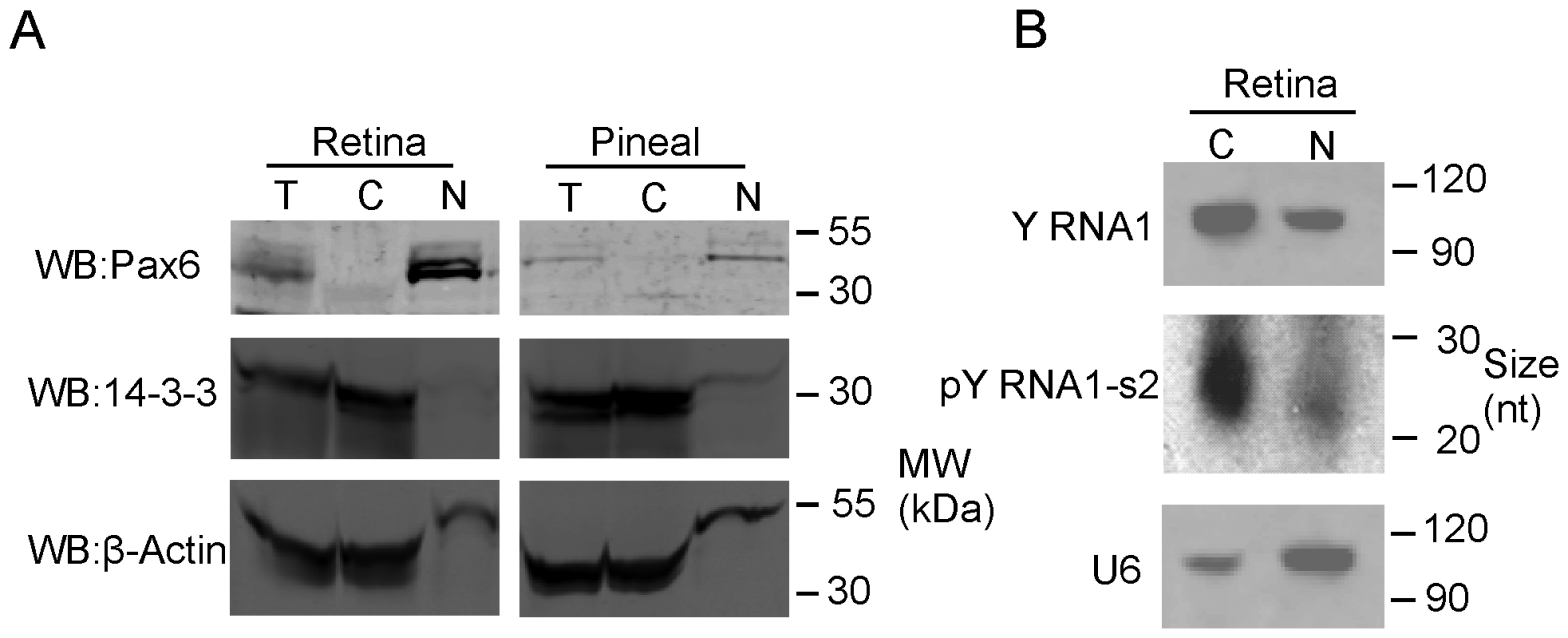
Cellular distribution of Y RNA1 and pY RNA1-s2. **A.** Retinal tissue was obtained from three animals, pooled and dissociated in lysis buffer with the addition of RNAse inhibitors, as described in “Materials and Methods”. These preparations were pooled and partitioned into nuclear and cytoplasmic fractions. Equal proportions of each fraction were subjected to SDS-PAGE and western blotted for Pax 6 (nuclear fraction control), 14-3-3 (cytoplasmic control) and β-Actin (cytoplasmic control). **B.** Northern blot of total RNA extracted from cytoplasmic and nuclear fractions as described above, separated on a denaturing 15% TBE-Urea PAGE gel, using a probe directed toward pY RNA1-s2, indicating the predominance of pY RNA1-s2 in the cytoplasm, but also detectable in the nuclear fraction. The panel indicated by “Y RNA1” shows the un-processed Y RNA and the panel labeled “pY RNA1-s2” shows the processed Y RNA1 that migrates at ∼27 nt. The blot was re-probed with a probe directed to U6 (as a control) indicating the majority is present in the nucleus.

To determine the subcellular location of processed pY RNA1-s2, cytoplasmic and nuclear protein and RNA extracts were prepared from rat retina and pineal tissues (Figure 5). Pax6 and the small nucleolar RNA U6 were used as markers for the nuclear fraction (Figure 5A; upper panel, Figure 5B; lowest panel) and 14-3-3 and β-actin were used as markers for the cytoplasmic fraction (Figure 5A; lower panels, Figure 5B; upper panels). Northern blot analysis revealed that the un-processed and processed Y RNA1 is enriched in the cytoplasm and detectable at lower levels in the nucleus (Figure 5B).

### pY RNA1-s2 binds selectively to Matr3 and other nuclear proteins

**Figure 6:**
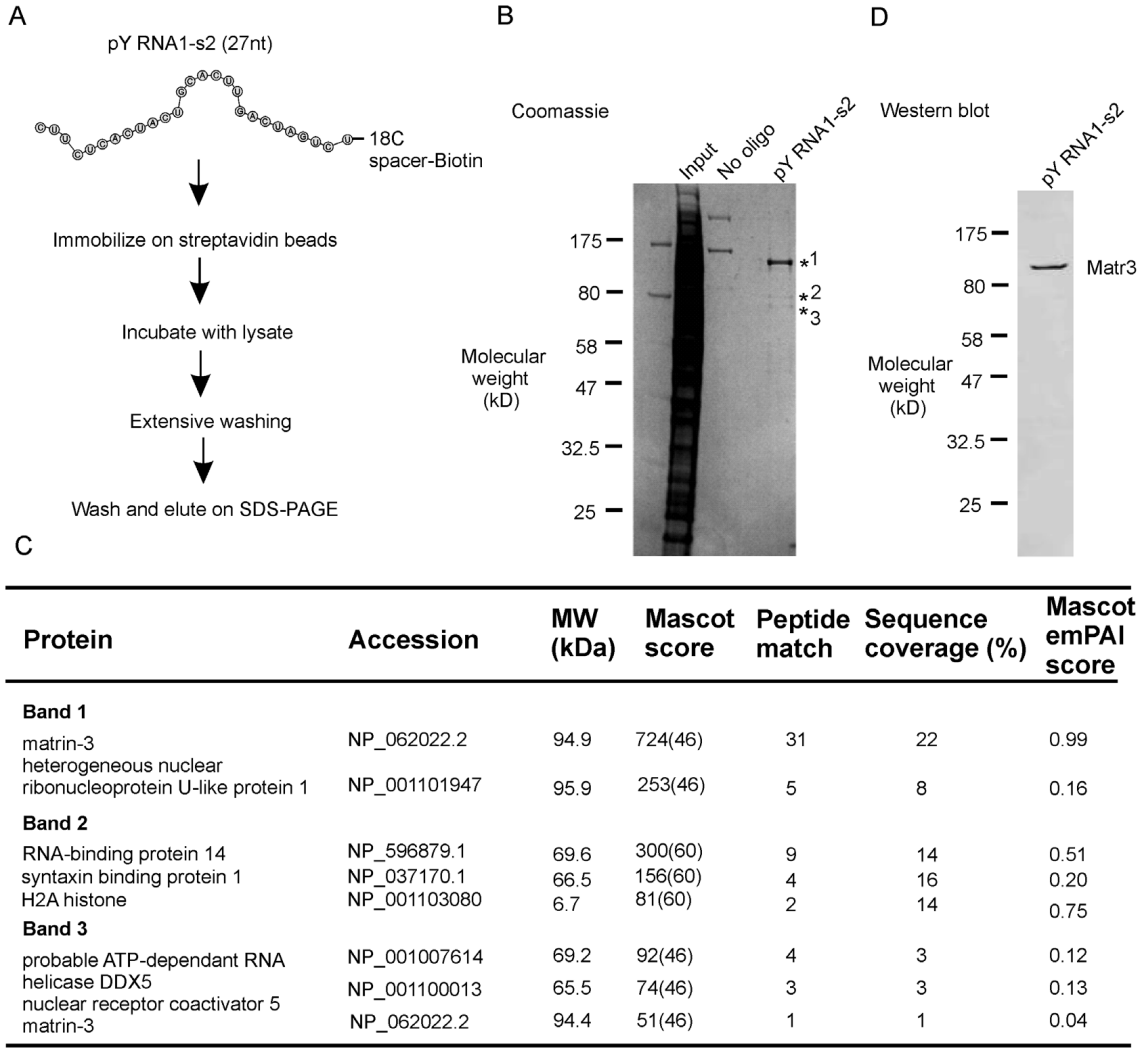
Identification of pY RNA1-s2 interacting proteins by affinity chromatography and mass spectroscopy. **A.** Scheme of the RNA:protein complex isolation procedure used in this investigation: a biotinylated RNA oligonucleotide corresponding to pY RNA1-s2 was synthesized with an 18C spacer, followed by a Biotin moiety at the 3′ terminus. The pY RNA1-s2 probe was coupled to streptavidin beads and incubated with lysate prepared from a pool of six rat retinas, followed by extensive washing and elution by SDS-PAGE. **B.** Coomassie blue staining shows pY RNA1-s2 predominantly associates with a protein migrating at ∼120 kDa, indicated with *1. Two minor bands are also present - indicated with *2 and *3; details in the table below. **C.** Table summarizing LC-MS/MS analysis of pY RNA1-s2-bound proteins. Mascot results were filtered for significance with p<0.05 and an identity score cut-off value set to report only ion scores with extensive homology. Results are sorted by best Mascot score. Key: *Protein* – Name of the protein in indicated band, *Accession* – the RefSeq protein Accession number, *MW* – Molecular weight in kDa, *Mascot score* – Probability score calculated by the Mascot search algorithm – parenthesis indicates the value at which the mascot score reaches identity, *Peptide match* - number of significant peptides recorded for each protein, S*equence coverage (%)*: sum of amino acids in matching peptides divided by the total number of amino acids in the protein, *Mascot emPAI score*: provides a measure of protein abundance. See “Materials and Methods” for a full explanation of how the parameters are calculated. **D.** Confirmation of Matrin3 association with pY RNA1-s2 by western blot, revealed by repeating the experiment performed in **A**, and using Matr3 antiserum (described in “Materials and Methods”).

The results of affinity chromatographic purification of pY RNA1-s2 binding proteins in retinal tissue and LC MS/MS analysis are presented (Figure 6, “Materials and Methods”). Band 1 was identified as Matr3, with 31 unique peptides (Figure S4 in File S1). In addition, five peptides identified from the same band correspond to another protein: heterogeneous nuclear ribonucleoprotein U-like protein. Peptides isolated from the minor bands migrating at ∼55 kDa (indicated in Figure 6B) correspond to Matr3, syntaxin and other nuclear proteins with RNA binding domains. Two non-specific bands are apparent in the “No oligo” lane (Figure 6B) and were identified as myosin (data not shown). The identity of major protein in the eluate as Matr3 was confirmed by immunoblotting (Figure 6D); anti- hnrpul1 antibodies were not available for confirmation of the mass spectroscopy data (data not shown).

To approximate the ratio of Matr3 to hnrpul1, the number of peptides detected for each protein, was normalized to the number of theoretical tryptic peptides (see “Materials and Methods” for a full description) to calculate an “emPAI” score (exponentially modified Protein Abundance Index) [25]. The results of this approximation indicate that Matr3 is at least 5 times greater than hnrpul1 (Figure 6C, compare 0.99 with 0.16). It should be noted that although it is clear that both proteins are present in the eluate, this quantitation used is imprecise because of potential differences in the ability of the method to detect tryptic peptides derived from each protein; accordingly, the resulting ratio can only be considered as rough approximation.

### Both RRM domains of Matr3 are required for selective binding of pY RNA1-s2

**Figure 7:**
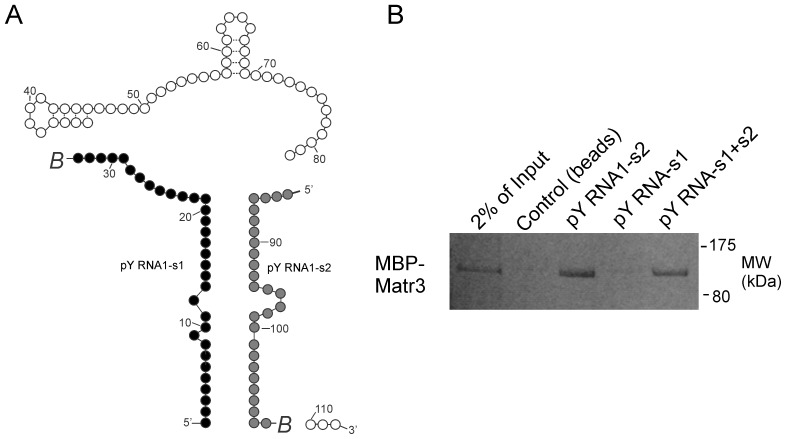
pY RNA1-s2 selectively binds to Matr3. **A.** Schematic depicting the two regions of Y RNA used in a binding assay with a Maltose Binding Protein fusion with Matr3 (MBP-Matr3). Biotin groups, indicated with *italic B*, were chemically attached to the 3′ end of each oligo. pY RNA1-s1 is indicated with black circles and pY RNA1-s2 with grey circles. **B.** the RNA oligonucleotides pY RNA1-s1, pY RNA1-s2 or an equimolar mix of both oligos (pY RNA1-s1+s2) were coupled to streptavidin beads, incubated with MBP-Matr3 and then washed extensively. A lysate prepared from six rat retinas was subsequently incubated with the beads, which were then washed; the proteins associated with the beads were recovered into sample buffer, resolved by SDS-PAGE and detected by Coomassie staining (details are available in the “Materials and Methods”).

pY RNA1-s1 contains an imperfect antisense nucleotide sequence with pY RNA-s2, reflecting the base pairing required for formation of the stem in Y RNA1. We therefore investigated the longer pY RNA1-s1 using the same binding assay approach described in Figure 6, with the exception that a maltose binding protein fusion of Matr3 was used (MBP-Matr3; Figure 7B). The biotin-tagged oligos were used for the binding assay (Figure 7A). In contrast to the strong interaction of pY RNA1-s2 and Matr3, there was no interaction with pY RNA1-s1 and Matr3 (Figure 7B, lane 4). To investigate the possibility of Matr3 binding double stranded pY RNA1 RNA oligonucleotides, pY RNA1-s1 and pY RNA1-s2 were mixed in equimolar ratios, heated and cooled to anneal the oligos, then incubated with MBP-Matr3 as above. The annealed oligos show reduced binding to Matr3 (Figure 7B, lane 5) relative to that of pY RNA1-s2 alone.

**Figure 8:**
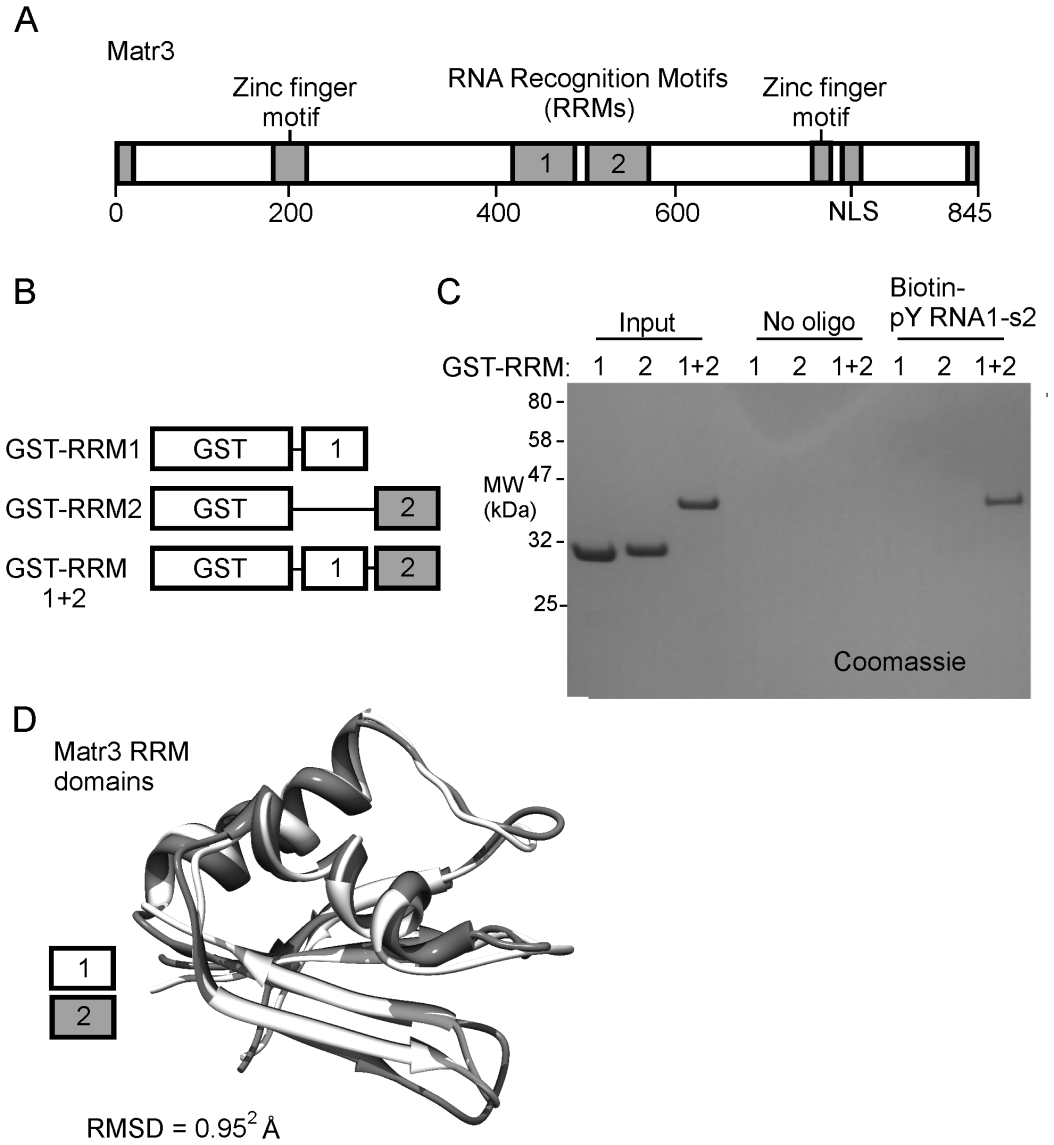
Both RRM domains of Matr3 are required for the interaction with pY RNA1-s2. **A.** Schematic of Matr3 indicating the Zinc finger binding domains and the RNA Recognition Motifs (RRMs) from within Matr3. **B.** Schematic of constructs created as N-terminal Glutathione S-transferase (GST) fusion proteins with the RRM from Matr3, used in this binding study. **C.** Immobilized pY RNA1-s2 was incubated with the indicated GST-RRM proteins and SDS-PAGE followed by Coomassie blue staining; this demonstrates that RRM1+2, but not RRM1 or RRM2 alone bind to pY RNA1-s2. “Input” lanes represent the total amount of each protein (2 µg) that was incubated with either beads alone (no oligo lanes) or with 100 pmols of pY RNA1-s2-biotin pre-coupled to streptavidin M280 beads (Biotin-pY RNA1-s2 lanes). **D.** Alignment of crystal structures of the Matr3 RRM domains (PDB ID: 1WEX and 1X4F) obtained from the RCSB database [39] reveal an RMSD of 1.03 Å.

Matrin3 has two conserved RRMs (Figure 8A), each of which binds RNA [26], [27]. Here we found that neither of the Matr3 RRMs exhibited a strong affinity for pY RNA1-s2 (Figure 8C) when tested individually; however robust binding was observed to occur with a construct containing both RRMs connected by the native inter-domain linker region (Figure 8C, far right lane).

### Identification of the nucleotide bases critical for pY RNA1-s2:Matr3 binding

**Figure 9:**
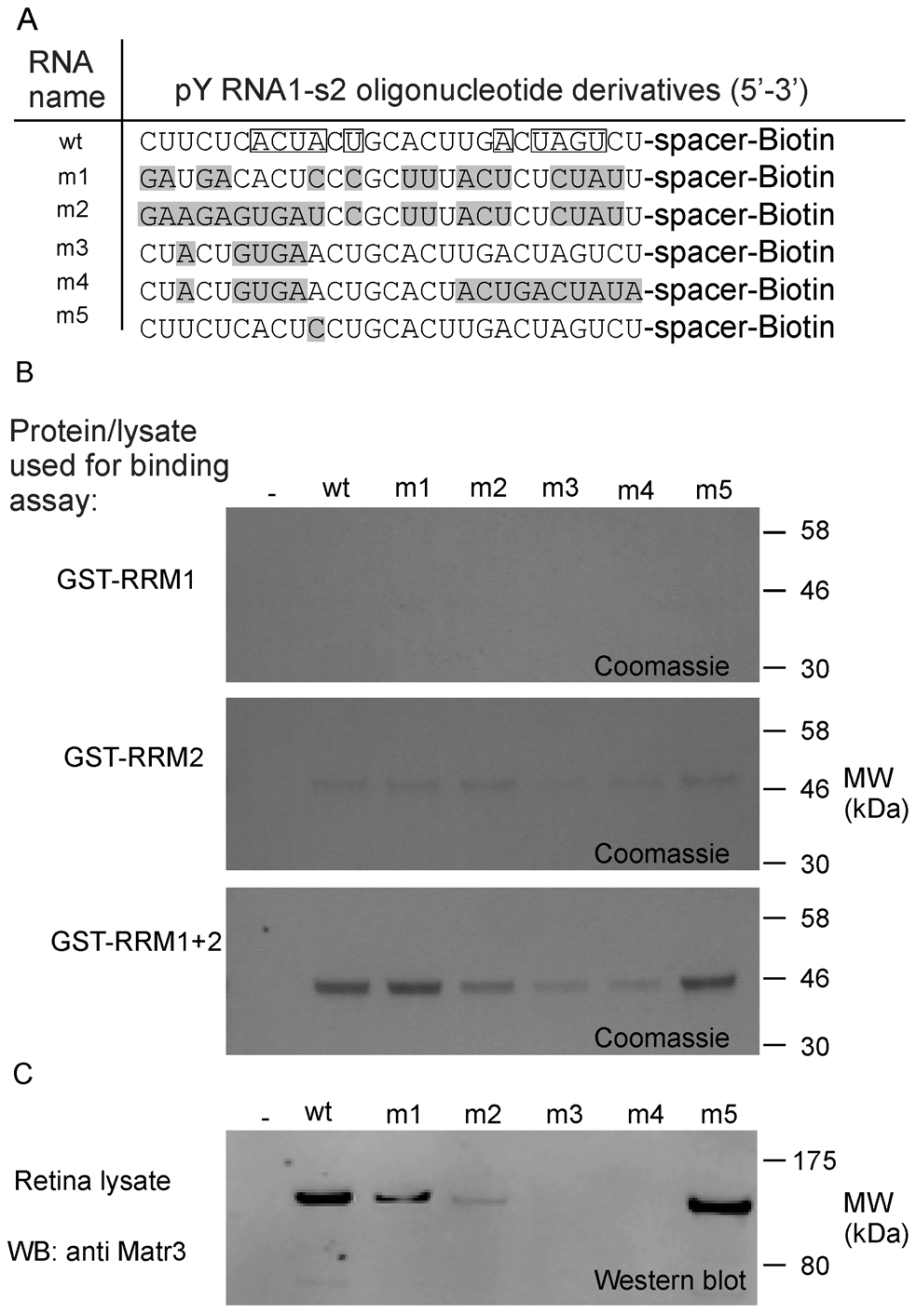
Isolated RNA recognition domains and endogenous Matr3 bind to pY RNA1-s2 in a sequence specific manner. **A.** Details of RNA oligonucleotides used to examine the interaction with Matr3. Mutated bases with respect to wild type (*wt*) are indicated with grey shading (*m1*, *m2*, *m3*, *m4* and *m5*). “Spacer” indicates an 18C spacer moiety attached to the 3′ end of the RNA, terminated with Biotin. Boxed residues indicate a pseudo-palindrome sequence. **B.** Derivatives of Matr3 conjugated with GST were incubated with the indicated RNA oligonucleotide. The constructs are detailed in Figure 8B and comprise: GST-RRM1, GST-RRM-2 and GST-RRM1+2 respectively. The interaction was examined by Streptavidin conjugated magnetic bead pull-down experiments: the bound protein is revealed by SDS-PAGE analysis followed by Coomassie blue staining. **C.** Matr3 is identified by western blot, revealing the interaction of pY RNA1-s2 and mutants with native Matr3. Lysate prepared from rat retina was incubated with the same pY RNA1-s2 mutants as used in **B**.

A panel of pY RNA1-s2 mutants was produced to explore the possibility of critical nucleotides necessary for the interaction with Matr3. Mutant pY RNA1-s2 oligos, referred to as m1 through m5 (Figure 9A), were coupled to Streptavidin beads (as described in “Materials and Methods”) and incubated with different sources of Matr3. Peptides containing the RRM domains were investigated using the same affinity methods described above and were analyzed by SDS-PAGE followed by Coomassie staining (Figure 9B). The isolated RRM subunits did not associate to any significant extent with the wild type pY RNA1-s2 or any of the mutant oligos (Figure 9B), consistent with data in Figure 7B. The construct containing both RRM domains in a single construct interacted robustly with the wild type pY RNA1-s2 (wt1), the scrambled pY RNA1-s2 (m1) and the oligo containing a point mutation at position 10: Adenosine mutated to Cytosine (m5). The m3 and m4 mutations decreased the interaction markedly. Interestingly the m3 mutant has only five mutations that are located toward the 5′ end of the RNA oligonucleotide. The sequences of the mutated oligos do not exist in the rat genome (rn4), as verified by a BLAST search (data not shown).

The immobilized mutated RNA oligos (m1, m2, m3, m4 and m5) were incubated with extracts of retina to test their ability to associate with Matr3; Matr3 was then detected by Western blotting. The scrambled mutant (m1) reduced the association by ∼50% (Figure 6B, lower panel). Consistent with binding studies using recombinant Matr3 RRM domains, above, mutants with targeted changes to the 5′ and 3′ regions of pY RNA1-s2 had a more pronounced effect on the interaction (Figure 6B). Mutations in the 5′ end of the RNA had the most striking effect of completely abrogating the association with Matr3 (m3 and m4).

### Phosphorylation enhances association of Matr3 and pY RNA1-s2

**Figure 10:**
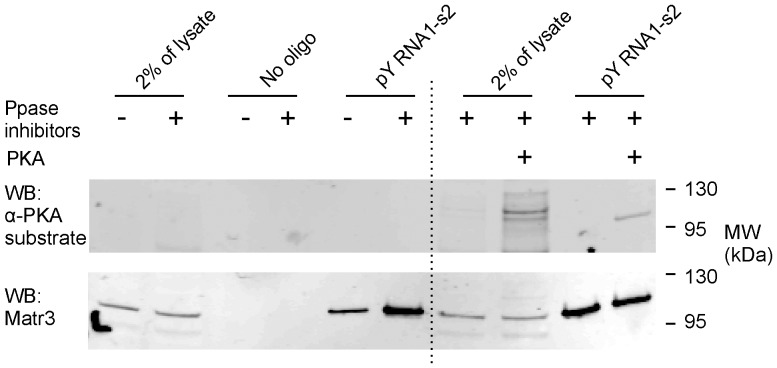
Phosphorylation enhances the interaction between Matr3 and pY RNA1-s2. Pre-conjugated biotinylated pY RNA1-s2: streptavidin beads (indicated with “pY RNA1-s2”) were incubated with retina lysates prepared from six animals, as described in “Materials and Methods”. Negative control binding assays are labeled “control”. Load controls are indicated with “2% of lysate”. The recovered proteins were subjected to SDS-PAGE and western blotting using an antibody raised against a generic phosphorylated substrate (α-PKA substrate) followed by re-probing against Matr3. Prior to the binding assay, retina lysates were prepared in the absence (−) or presence (+) of phosphatase inhibitors (Ppase inhibitors) or recombinant protein kinase A (PKA), to enhance phosphorylation of endogenous Matr3. See “Materials and Methods” for details of inhibitors and PKA used. The lower panel shows a re-probe using an anti-Matr3 antibody of the upper blot.

Matr3 is a substrate for several protein kinases [28], including PKA [29]. The impact of Matr3 phosphorylation on the binding to pY RNA1-s2 was investigated first by analysis of retina lysates prepared with or without broad spectrum protein phosphatase inhibitors and subsequently using recombinant protein kinase A (PKA) with phosphatase inhibitors prior to the binding assay (Figure 10). The phosphatase inhibitors preparation (see “Materials and Methods”) target serine/threonine and tyrosine phosphatases. The inclusion of phosphatase inhibitors did not increase the signal detected by the anti-PKA substrate antibody (Figure 10, upper panel), but did enhance the amount of Matr3 associated with pY RNA1-s2 (Figure 10, lower panel). Inclusion of PKA in the retina lysate produced several immuno-reactive anti-PKA substrate bands, with an intensely phosphorylated band corresponding in size to Matr3 (Figure 10, upper panel). PKA-dependent phosphorylation of Matr3 increased the level of pY RNA1-s2 binding, to the level of that produced by the inclusion of phosphatase inhibitors (Figure 10, lower panel).

### Retinal Matr3 exists in both nucleus and cytoplasm

Previous studies have established that Matr3 is a nuclear protein. Here we found that the distribution of Matr3 in the retina is limited primarily to the nucleus (Figure S5 in File S1), although Matr3 was also clearly detected in the cytoplasm, albeit at lower levels. Moreover, the cytoplasmic level in the retina was found to be higher than that in the pineal gland.

## Discussion

The results of these studies have clearly identified two new small RNAs, pY RNA1-s1 and pY RNA1-s2, and have identified a specific binding partner for one. In addition, these studies have expanded our understanding of tissue and cellular distribution of pY RNA1-s2. These results will be discussed below.

### Characteristics and distribution of pY RNA1-s1 and pY RNA1-s2

pY RNA1-s1 and pY RNA1-s2 cannot be considered to be miRNA because of two sets of observations. One is that the smallest read that aligned to Y RNA1 is pY RNA1-s2 and at 27 nt is longer than any known miRNA [20]; the second is that it is processed from a known small RNA, inconsistent with the known mode of miRNA production [30]. A thorough investigation into the highly related Y5 and Y3 RNA fragments did not reveal interaction with the miRNA pathway [16], lending weight to the probability that Y RNA fragments are independent of miRNA biology. It has been reported that Y RNA is processed into smaller fragments in other cells types, including solid tumors [31] and cell lines in response to induced apoptosis [23]. A small RNA product from the human Y RNA 3 homolog has been investigated for miRNA-like silencing activity, but no evidence was found [31]. The sequence identified herein is processed from a different Y RNA (Y RNA1) and therefore miRNA/siRNA-like silencing activity cannot be ruled out, but is unlikely – as discussed further below.

It is interesting to note that the most conserved region of Y RNA1, the main part of the stem loop present within all Y RNA genes, is the sequence processed into pY RNA1-s1/2 (Figure 1). Recently, stem-bulged RNAs (sbRNAs) were identified as nematode homologs of vertebrate Y RNAs [32] suggesting deeply conserved evolution. This evolutionarily conserved region may have a function involving binding similar proteins in diverse organisms.

Our studies on the tissue distribution have provided evidence that abundance of pY RNA1-s2 in the retina is 20-fold greater than in other tissues (Figure 2). It is not clear whether this is due to greater capacity to process the parent or if pY RNA1-s2 has a greater stability in the retina due lower levels of degradation and/or higher protein binding; other factors might also be involved, although random degradation of Y RNA1, particularly given the known efficiency of RNAses (reviewed in [33]) present in all cell types, appears unlikely. Whereas the underlying mechanism(s) underlying the 20-fold higher level of pY RNA1-s2 remains to be determined, the robustly greater abundance in the retina points to the possibility the pY RNA1-s2 might have a specific function in the retina not seen in other tissues. The presence of pY RNA1-s2 in all retinal layers may indicate that this level of expression is first established in the precursor cells that give rise to all retinal cells [34], [35].

Retinas obtained from several vertebrates revealed the presence of pY RNA1-s2 (Figure 4), suggesting a conserved function in this tissue. This confirms phylogenetic analysis that reports the preservation of the Y RNA cluster throughout tetrapod evolution [9], [14]. Our study reveals a similar pattern of expression between pY RNA1-s2 and Y RNA1. It is interesting to note the apparent lower expression level of Y RNA1 compared to pY RNA1-s2. This is most likely due to differences in the secondary structure of pY RNA1-s2 and Y RNA1 that affect the efficiency of the reverse transcription step in qRT-PCR.

In situ hybridization of retina sections was performed using an LNA probe directed toward pY RNA1-s2 (Figure 3A). However, it is worth noting that the probe cannot differentiate between the un-processed Y RNA1 and the cleaved pY RNA1-s2. Therefore the staining will represent both processed and un-processed Y RNA1. Intense staining reveals the majority of expression in the GCL and PRL.

To explore the relative levels of pY RNA1-s2 and Y RNA1 in each section, three layers were separated using laser capture micro-dissection and the recovered RNA subjected to qRT-PCR (Figure 3C). This analysis shows that the PRL has the highest level of pY RNA1-s2 and Y RNA1, the same as the in-situ hybridization experiments (Figure 3A). However, this is due at least in part to photoreceptors representing approximately 70% of the total retinal cell population [18]; using other small RNAs (U5, U6 and Sn202) for normalization, it is apparent that the relative level of pY RNA1-s2/Y RNA1 is greater in the GCL than in any other retinal layer. The expression of pY RNA1-s2 in multiple retinal layers was also investigated in mouse retina, with similar conclusions. The indication that pY RNA1-s2 is present in all cell layers of the retina points to the possibility that it functions through a similar mechanism in cells in the PRL, INL and GCL to control RNA biology.

### pY RNA1-s2 selectively binds to Matr3

The results of pY RNA1-s2 binding experiments presented here appear to confirm the hypothesis that pY RNA1-s2 has a specific binding partner (Figure 6B), as do other small RNAs. Specifically, there appears to be relatively selective binding of this molecule to Matr3 (Figure 9). This discovery, which is based on MS/MS results and immunostaining (Figure 6D) argues that pY RNA1-s2 may function through interactions with this protein. It is known that Matr3 has a predominantly nuclear localization [36], [37] and is considered part of the nuclear matrix. However, several reports indicate Matr3 is also located in the cytoplasm [27]. Our investigations confirmed that Matr3 is primarily a nuclear protein in retina, and that the cytoplasm component is relatively minor (Figure S5 in File S1). In contrast, we found an almost exclusive nuclear localization in the other tissues examined; the pineal and pituitary gland (Figure S5 in File S1). This raises the possibility that retinal Matr3 has a special function in the retina that is localized in the cytoplasm.

The localization in retina of pY RNA1-s2 follows that of the un-processed Y RNA1 and is primarily cytoplasmic (Figure 5). The subcellular location of Matr3 would presumably have a great impact on the function of pY RNA1-s2 as a Matr3 client. The findings presented here suggest that Matr3 and pY RNA1-s2 could interact in both the cytoplasm and the nucleus, where they coexist.

Matr3 appears to bind pY RNA1-s2 through specific interactions with the two RRMs in the protein. RRMs frequently occur in pairs, or larger multiples, but multiple RRM domains are not necessarily required for RNA binding (reviewed in [38]). The amino acid sequence of the Matr3 RRM domains share little sequence similarity (Figure S6 in File S1), but are remarkably similar at the structural level (RMSD<1 Å), as revealed by an alignment of the crystal structures obtained from the Protein Data Bank [39] (Figure 8D). The Matr3 domains RRM1 and RRM2 are separated by a 22 amino acid inter-domain linker; therefore each RRM could feasibly act in concert to cooperatively bind the same molecule of pY RNA1-s2. This interpretation is supported by the finding of an absolute requirement of both Matr3 RNA binding domains for the association with pY RNA1-s2 (Figure 8).

Matr3 associates predominantly with single stranded pY RNA1-S2, as revealed by an *in vitro* binding assay that compared pY RNA1-s1, which contains an almost complimentary sequence to pY RNA1-s2 (to form the stem region of Y RNA), with pY RNA1-s2 (Figure 7). Performing an identical binding assay, with both RNA oligonucleotides pre-annealed reduced binding to Matr3, indicating that Matr3 has the ability to discern single stranded RNA from double stranded RNA, utilizing both RRM domains.

Mutation of pY RNA1-s2 oligonucleotides, chosen to probe potential sequence preferences, identified at least 5 bases critical for the interaction; mutation of bases in the 5′ region of pY RNA1-s2 completely abrogated the interaction (Figure 9). The selective nature of Matr3 binding suggests a specific repertoire of transcripts, perhaps playing a role in post transcriptional regulation. Indeed, Matr3 appears to play a number of major roles in cellular function, as knock-down studies involving siRNA against Matr3 have shown disruption of the cell cycle [28], reduced mRNA stability [40] and effects on cell proliferation [41].

Our finding that phosphorylation of Matr3 enhances binding is consistent with a number of reports in the literature indicating that the protein can be phosphorylated [28], [29], and is the first to indicate that phosphorylation alters association of Matr3 with a binding partner. Our discovery that phosphorylation changes the affinity of Matr3 for pY RNA1-s2 is of potential interest on two levels. It indicates that the function of Matr3 as an RNA binding protein is subject to cAMP-dependent phosphorylation and that Matr3 phosphorylation is one part of a broader effect of cAMP on cell metabolism. On another level, one that involves pY RNA1-s2, it would appear that phosphorylation enhances the binding affinity of pY RNA1-s2 to Matr3 (Figure 10).

### Summary

Our study has revealed that Y RNA1 is processed in the retina to produce a previously unrecognized ∼27 nt RNA, pY RNA1-s2 that binds specifically to Matr3. Matr3 may function as an integrator of transcriptional regulation, considering the location of Matr3 in the nucleus, and the many described roles in maintenance of DNA integrity and involvement in RNA biology. The notably high level of pY RNA1-s2 in the retina and high conservation suggest it plays a role in retinal biology, possibly through binding to Matr3 and that through this interaction it influences RNA biology. It is reasonable to consider that the pY RNA1-s2/Matr3 interaction is an essential element of vision. Hence, the findings of this study have potential translational value in understanding the pathophysiology of the eye and in the design of drugs to treat eye diseases.

## Materials and Methods

### Animals and tissues

Ethics statement: Animal use and care protocols were approved by NIH or Emory Institutional Animal Care and Use Committees and followed the guidelines of the National Research Council's Guide for Care and Use of Laboratory Animals (Vol. 8) [42] and the Animal Research: Reporting In vivo Experiments (ARRIVE) guidelines.

Rodents and chickens used for these studies were provided food and water were provided ad libitum in rooms with automatically controlled lighting which provided 200 to 400 lux during the day; the lighting schedule was 14∶10 light∶dark (L∶D) for rats and 12∶12 LD for mice and chickens. Animals entrained to these lighting cycles for at least on week prior to euthanasia. Euthanasia was done during the day at Zeitgeber times (ZT) 0700 to 0800 or at night at ZT 1800 to 1900 under dim red light. Rats (Sprague Dawley, female, 180 to 250 grams; Taconic Farms Inc., Germantown, NY) were euthanized by carbon dioxide inhalation; tissues were obtained from groups of 10 to 40 animals. Mouse tissues were obtained from two sources. One was a 6 week old male C57BL/6 mouse maintained at the NIH; euthanization was by carbon dioxide inhalation; the second was a group of one year old C57BL/6J-*Pde6b^rd1-2J^*/J (rd1) mice and C57BL/6 mice maintained at Emory University; euthanasia was by cervical dislocation three pools of retinal tissue were prepared, each containing equal amount of male and female tissues. Chicken retinal tissue was obtained from an eight day old male (White leghorns, HyLine International, Covington, GA) following euthanasia by decapitation.

Rhesus macaques (three adult males, 8 to 10 years of age) were obtained from the NIH recycle program; care and use follows National Research Council guidelines (1). Animals were housed in pairs or groups of compatible individuals unless they are exempted from pair or group housing for scientific or medical reasons or due unsuitable individual traits (e.g. excessive aggressiveness etc.), in which case they were housed in single cages. Animals were housed in stainless steel cages, sized to the weight of the animals as detailed (Table 3.5 of [42]) in rooms housing up to 40 individuals. Each cage had a perch shelf. For animals which are pair housed or group housed dividers between adjacent cages were removed to increase total size of accessible area. Animal cages were hosed daily. Floors of animal rooms were disinfected and hosed daily. Cages were sanitized in the cage washers every 2 weeks. Animals were fed twice daily with commercial scientifically formulated primate diets. The amount of provided food depended on the animals' weights. Additionally, animals received fresh fruits and/or vegetables at least 3 times per week. Animals received municipal water ad libitum via automatic watering devices. The lighting schedule in animal rooms was L∶D: 1∶12; temperature was maintained between 22 and 26°C; humidity was maintained between 30 and 70% with 10 to 15 fresh air changes per hour. In addition to pair- and group housing, environmental enrichment was achieved through elaborate animal enrichment program including provision of toys, mirrors, TV animal shows and frequent interactions with veterinary care staff. Animals were monitored at least twice daily to insure psychological and physical well-being. Prior to euthanasia, animals were briefly immobilized in their cages using a squeeze mechanism, anesthetized by intramuscular administration of Ketamine (10 mg/kg), followed by a rapid administration of a lethal dose of Euthanasia Solution. (http://www.vedco.com/index.php?option=com_content&task=view&id=323&Itemid=26) at 100 mg/kg of Sodium Pentobarbital. Tissue was rapidly removed, placed on Dry Ice and stored at −8O°C.

Adult ewes (outbred Dorsett×Hampshire mix; 150 to 200 pounds,) were purchased from a commercial source of market sheep (September 1995) and housed for two weeks in groups of two to three in covered outdoor pens (∼1000 f^2^) at the NIH Animal Center. Animals received natural lighting during the day (L∶D approximately 12∶12); dim white light was provided during the night, as required for security. Water and hay were provided ad libitum; a daily grain supplement was also provided on a weight basis. Animals were monitored several times a day for signs of illness… Animals were not used for any purpose other than for collection of tissues for this investigation. For euthanasia, animals were transferred (1200 to 1300 hours) to an indoor room that was isolated from other animals and administered a lethal I.V. bolus of Somlethal® (25 ml/100 kg). Tissue was rapidly removed, placed on Dry Ice and stored at −8O°C.

Bovine retinal tissue was removed from eyes delivered on wet ice by a local abattoir (J. W. Trueth & Sons, Baltimore, MD), placed on Dry Ice and stored at −8O°C

Human tissues were purchased from a commercial repository (National Disease Research Interchange, Philadelphia, PA). Samples and donors were coded as required for deidentification. The tissues were obtained with informed consent provided by the donors or by their next of kin. Under United States Department of Health and Human Services human subjects regulations (45 CFR Part 46), use of such samples is not considered human research and ethics review is not required. The donors (2 males and 1 female) ranged in age from 28 to 62, the time-of-death was between 11:10 am and 12:30 pm and the post-mortem interval to tissue removal was 6.5 to 8 hours. The samples (whole eyes or posterior poles) were delivered on wet ice within 24 hours post-mortem; retinas were dissected, placed on Dry Ice and stored at −8O°C.

### Laser capture microdissection (LCM)

Whole female rat eyes, embedded in optimal cutting temperature compound (OCT; Sakura Finetek, Torrance, CA) and frozen in −80°C, were used for LCM. Rat eye sections were made at 12 µm thickness and mounted on polyethylene naphthalate membrane glass slides (Applied Biosystems, Foster City, CA). The sections were stained with HistoGene staining solution (Applied Biosystems), dehydrated in graded ethanol solutions (75%, 95%, 100% ethanol), and cleared in the xylene solution. LCM was performed to microdissect the GCL, INL, and PRL (which included both the outer nuclear layer and inner segments of photoreceptors) onto HS CapSure non-contact LCM films using the ArcturusXT system (Applied Biosystems). RNAqueous-Micro Kit (Life Technologies) was subsequently used to purify both large and small RNA species from the captured tissue. For each retinal cell layer, a pool of three samples was prepared.

### Biochemical Methods

#### Small RNA sequencing and bio-informatic analysis

The purification of pineal RNA and sequencing have been described [17]. FASTQ files have been deposited at the NCBI short read archive (SRA; accession number: SRA049977). The reads were aligned to the rat genome (rn4) using Novoalign version 2.06.09 with the following parameters: -a TCGTATGCCGTCTTCTGCTTG –m –l 17 –t 60 –s 1 -r ALL. Details of each parameter are found at www.novocraft.com. Briefly, these settings afford the possibility for each read to be reported in multiple genomic locations, if the alignment quality is within 5 points of the best alignment. The chosen settings can be considered ‘less stringent’ and allow 2 mismatches per read. The consensus sequence for pY RNA1-s2 is: 5′-CTTCTCACTACTGCACTTGACTAGTCT-3′, as determined using the alignment described above and maps to a predicted Y RNA in the Ensembl database (Gene: ENSRNOT00000053328.1). A BLAST search of Genbank [43], [44], restricted to sequences from the taxonomic id 10114 (Rattus), indicated partial matches to known transcripts: U84683.1 and X69719.

The number of times each read mapped to the rat genome was parsed from the output SAM file, using the optional multiple mapping tag: ZN (using a custom Python script) and grouped into three bins that span Y RNA (outlined in Figure 1). The number of mapping locations were sorted into bins and reported in Table 1. The sequences were also blasted against piRNA bank (http://pirnabank.ibab.ac.in/index.shtml); no hits were identified.Y.

#### Northern blotting of Y RNA-derived small RNA

Approximately 10 µg of total RNA was extracted from the tissues using mirVana columns (Life Technologies, Carlsbad) following manufacturer's instructions and subjected to denaturing polyacrylamide gel electrophoresis (PAGE) on a 15% TBE (Tris-Borate-EDTA)-urea gel (Life Technologies) and transferred to Nytran Hybond membrane (GE, Piscataway). The small RNAs were cross-linked to the membrane using 1-ethyl-3-[3-dimethylaminopropyl] carbodiimide hydrochloride [7]. The membrane was processed using the reagents from the Nucleic Acid Detection Kit (Pierce, Rockford) and then incubated with a Locked Nucleic Acid (LNA) probe directed against pY RNA1-s2. The sequence of the probe used is: 5′-Biotin-CTA+GTC+AAG+TGC+AGT+AGT+GAG+AAG-3′; LNA residues are indicated by the “+” sign (Exiqon, Denmark). The signal was detected using streptavidin-HRP (horse radish peroxidase) and enhanced chemo luminescence (ECL). The DNA probe (Idt, TX) used to detect U6 has the sequence: 5′-Biotin-GAATTTGCGTGTCATCCTTGCGCAGGGGCC-3′.

### In Situ Hybridization of pY RNA1-s2/Y RNA1

The retinas from P25 rats were fixed by incubating with 4% paraformaldehyde (PFA) in PBS at 4°C overnight, washed in PBS, equilibrated with 20% sucrose in PBS for 24 hr, and embedded in OCT (ThermoFisher, CA). Ten-micrometer sections were cut on a cryostat, transferred on Superfrost plus/Colorfrost plus microscope glass slides (DAIGGER, Vernon Hills, IL), and stored at −80°C until use.

The slides were processed as indicated in [45], using the following probes: 5′-Dig-labeled LNA/DNA probe for pY RNA1-s2 (5′-AGACTAGTCAAGTGCAGTAGTGAGAAG-3′) was custom-ordered from Exiqon, Denmark and a control LNA/DNA probe (Cat#, 99004-01; 5′-GTGTAACACGTCTATACGCCCA-3′) was purchased ThermoFisher. After hybridization, slides were washed twice with PBS containing 0.2% Triton X-100 (PBST) for 5 min each and blocked in PBST containing 20% sheep normal serum at room temperature for 1 hr. Slides were incubated with sheep anti-digoxigenin, alkaline phosphatase (AP)-conjugated, sheep Fab fragment (1∶2,000 dilution; Roche) in the same blocking buffer at 4°C overnight. After three washes in PBST for 10 min each, slides were equilibrated twice for 10 min each with AP buffer (100 mM Tris (pH 9.5), 100 mM NaCl, 50 mM MgCl2, 0.1% Tween-20) containing 1 mM levamisole. Alkaline phosphatase (AP) substrate solution containing NBT (100 µg/ml) and BCIP (50 µg/ml) as well as 1 mM levamisole was applied to each slide for 4 hrs. The AP reaction was stopped by washing twice in PBS for 10 min each. Slides were incubated with DAPI in PBS for 3 min, washed once in PBS for 10 min, and mounted with a Flouro Gel mounting medium (Electron Microscopy Sciences) for microscopy.

### Affinity chromatography

Rat retinal tissue was collected from 40- to 60- days-old female and male rats, reduced to powder by crushing on solid CO_2_ and stored at −80°C. A 10 mg aliquot was homogenized using a polytron (Kinematic, Luzern, Switzerland) in lysis buffer (4°C) consisting of: 20 mM Tris, pH 7.4, 150 mM NaCl, 2 mM DTT, 0.5% NP-40 alternative (Calbiochem, San Diego), 40 U/ml RNasIN (Promega, Madison), 0.5 mM EDTA, 1× protease inhibitor (Roche, Indianapolis) and 2 mM MgCl_2_. The lysate was clarified by centrifugation at 16,000 g for 30 minutes at 4°C. Biotinylated RNA probes were synthesized by IDT (Iowa) and contained an 18 carbon spacer between the 3′ end of the RNA and the Biotin moiety. All RNA oligonucleotides were purified by HPLC and checked to be the correct molecular weight by electrospray ionization (ESI) mass spectrometry (IDT, Iowa). Details of the sequences used are shown in Figure 6A. One hundred pmol of each RNA oligo was pre-incubated with magnetic M-280 Streptavidin beads (Invitrogen, Carlsbad) for 2 hours before washing and subsequent addition to the lysate. The protein concentration was adjusted to 1 mg/ml before the addition of the Streptavidin:RNA complex. The RNA:bead complexes were incubated with lysates for 4 hours at 4°C, with gentle rotation. After 3 washes in high salt (0.5 M NaCl) lysis buffer, the final wash was in lysis buffer and elution of bound protein achieved by incubation in 40 µl 1× NuPage LDS (Lithium dodecyl sulfate) sample buffer (Invitrogen, Carlsbad).

### Analysis of phosphorylation and Matr3 binding to pY RNA1-s2

Protein lysates were prepared as described above, except with the addition of ‘broad spectrum phosphatase inhibitors’: 10 mM sodium orthovanadate (Na_3_VO_4_), 10 mM β-Glycerol phosphate (BGP) and 50 nM Microcystin-LR (MC-LR) (EMD Millipore, MA, USA). The concentration of the catalytic subunit cAMP-dependent protein kinase (Promega) was 100 u/ml, immediately prior to use. The lysis buffer was supplemented with 10 µM ATP and 100 µM MgCl_2_, freshly prepared and maintained at pH 8.0. Lysates were incubated at room temperature for 30 minutes, followed by cooling to 4°C. The binding assay was performed as described above.

### Analysis of bound proteins

RNA:protein complexes were denatured (90°C for 3 minutes in 1× loading buffer, (NuPAGE LDS, Invitrogen) and the supernatant was loaded onto 10% Bis-Tris NuPAGE gels (Invitrogen, Carlsbad) which were used for SDS PAGE. The resulting gels were either stained with Coomassie blue G250 according to Sambrook et al, detailed in [46] or transferred to PVDF membrane using the iBlot system (Invitrogen) according to manufacturer's instructions. Molecular weight was assessed using *Pre-stained Broad markers* (P7708S, NEB, MA, USA).

### Mass spectrometric analysis of proteins

The three most predominant Coomassie Brilliant Blue stained bands were excised, subjected to in-gel digestion using modified trypsin (Promega) and analyzed by two mass spectrometric methods. One portion of the peptide mixture was applied to a C18 column and analyzed on-line by an LTQ instrument (ThermoFinnigan, Waltham). Electron transfer dissociation (ETD) was used to achieve high resolution ms/ms spectra that were then searched against the rat NCBInr database using Protein Prospector software (Thermo Scientific, Waltham) and Mascot (Matrix Science, Boston). Another portion of the peptide mixture was concentrated by C18 ZipTips (Millipore, Bedford) and analyzed on a Voyager 5800 MALDI-TOF/TOF instrument. The resulting data files were searched against the rat genome (NCBInr) using Mascot, allowing up to two missed cleavages, oxidized methionine and alkylated cysteine residues.

### Western blot

Anti-Matrin 3 (part number A300-591A, Bethyl Laboratories, Texas) was used at 1∶2000. The anti-PKA substrate was used at 1∶1000 (part number 9621S, Cell signaling, California), was raised against the motif: RXXps/pS and can recognize phosphorylated substrates of PKA and PKC. Anti-Ro (Santa Cruz, California) and anti-hnrpul1 (part number H00011100-B01, Abnova, California) were used at 1∶1000. Anti 14-3-3γ (Prof A. Aitken, The University of Edinburgh) was used at a dilution of 1∶3000. Secondary antibodies conjugated to IR680 (Invitrogen, Carlsbad) or IR 800 (LI-COR, New Brunswick) dye were used at 1∶15,000 and 1∶5000 respectively and revealed using an Odyssey infrared imager (LI-COR, New Brunswick).

### Expression of Matr3 peptides

Three GST constructs were created encoding the first, second and both RRMs, termed GST-RRM1, GST-RRM2 and GST-RRM 1-2. These were cloned into pGEX-4T1 (GE, Piscataway) using the following primer sets: RRM1 primer set, *EcoRI* and *NotI* underlined, Fwd: 5′-GATCGGATCCCAAAAAGGCAGAGTGGAAACCAGC-3′ and reverse: 5′-GATCGCGGCCGCTTATTCTCTTATACTTCTGGGAT-3−Y. RRM2 primer set, *BamHI* and *NotI* underlined, Fwd: 5′-GATCGGATCCAAACCTGAAGGAAAACCAGAT-3′ and reverse: 5′-GATCGCGGCCGCCACCAGTTTTTTATATTTCTCAGA-3′. GST-RRM1-2 was produced using the appropriate combination of these primers. The constructs were transformed into *E. coli* BL21(DE3)pLysS and induced with 0.1 mM IPTG for 4 hours at 37°C. Pellets were re-suspended in 2× PBS (Phosphate Buffered Saline), 2 mM DTT and 1× protease inhibitor (Roche, Indianapolis), sonicated with three 30 second pulses (Bromwill, VWR, Bridgeport, NJ) followed by centrifugation before affinity purification using Glutathione Sepharose 4B (Amersham). GST-RRM1 and GST-RRM2 required only one-step purification to produce ∼98% pure protein (estimated from Coomassie blue staining). Following affinity chromatography with glutathione beads, GST-RRM1-2 required size exclusion chromatographic purification, performed using Superdex 200 in 1× PBS buffer containing 1 mM DTT and 0.5 mM EDTA. A subsequent ion exchange (MonoS) column (GE, Piscataway) was used with a 0 to 1 M NaCl gradient that resulted in a ∼98% pure preparation. A DNA construct encoding MBP-Matrin 3 (Dr Paul Kinchington, University of Pennsylvania) was expressed in NEBexpress bacteria (NEB, New England, USA) with 0.1 mM IPTG, supplemented with 2 mM glucose, shaking overnight at room temperature. The lysate was treated as above, before recovery with amylose resin and elution with 10 mM maltose. The eluate was further purified by size exclusion chromatography as above. The resulting preparation was ∼95% pure as estimated by Coomassie staining.

### Estimation of relative abundance of identified pY RNA1-s2-bound proteins

Raw data files from the LTQ mass spectrometer were converted to mascot generic format (mgf) before processing on the NIH Central Mascot Server searched against NCBInr database, using default parameters. The software calculates a simple score called emPAI (exponentially modified Protein Abundance Index [47]) that is calculated using the formula: 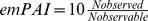
−1. N_observed_ is the number of peptides observed and N_observable_ is the number of potentially identifiable peptides – a number that reflects both the size and composition of the protein. The method is described at http://www.matrixscience.com and offers a convenient label free method to estimate relative protein abundance.

### Nuclear/cytoplasmic separation

Retina and pineal tissues were extracted and separated into nuclear and cytoplasmic fractions using a nuclear/cytoplasmic separation kit (BioVision, CA), following manufacturer's instructions. Briefly, 1 mg of retina and pineal tissues were gently dissociated in 200 µl of Cytosol Extraction Buffer A (supplemented with 10 U/µl RNAse inhibitors (RNaseIN, Promega)) using a dounce homogenizer at 4°C. Protein was determined using the BCA method (Pierce). RNA was extracted from the cytoplasmic and nuclear fractions using the miRVana RNA Extraction kit (ABI, CA) with a 1∶10 ratio of cellular extract to RNA lysis buffer provided in the extraction kit. The RNA concentration was determined by absorbance at 260 nm.

### Quantitative real-time polymerase chain reaction (qRT-PCR) for LCM

Retinal layer-specific transcripts (Brn3a (GCL), mGluR6 (INL), and Rho (PRL)) were quantified by quantitative real-time polymerase chain reaction (qRT-PCR) after the total RNA was reverse-transcribed to cDNA using QuantiTect Reverse Transcription kit (Qiagen, Valencia, CA). With 2 µL cDNA from each sample, qRT-PCR was performed in Bio-Rad iCycler (Bio-Rad Laboratories Inc., Hercules, CA) with a 25 µL total volume containing cDNA, QuantiFast SYBR Green PCR Master mix (Qiagen), and 1 µM forward and reverse primers for the gene of interest, and the fluorescence threshold value was calculated using MyiQ cycler software. The transcript level for each gene was normalized to the level of the housekeeping gene 18S rRNA and quantified according to the delta-delta Ct method [48]. PCR primers sequences were: Brn3a (Pou4f1) Forward (Fwd): 5′-ACTGGACCTCAAAAAGAACG-3′, Reverse (Rev): 5′-CAGACCTATTTGAAACGAACA-3′; mGluR6 Fwd: 5′-TGTTCCGCTCTTCCTCACTT-3′, Rev: 5′-GACCTTGGCTCACCGACT-3′; Rho Fwd: 5′-CACACCACTCAACTACATCCTGC-3′, Rev: 5′-GATTCTCCCCAAAGCGGAAG-3′; and 18S Fwd: 5′-GTTGGTTTTCGGAACTGAGGC-3′, Rev: 5′-GTCGGCATCGTTTATGGTCG-3′.

### Quantitation of small RNAs using polyA tailing RT synthesis and qRT-PCR

RNA was extracted from the corresponding LCM retina layers using miRVana (ABI) columns as described above. Due to the low yield of RNA, it was not possible to accurately determine the RNA concentration; therefore equal proportions of each sample were used for cDNA synthesis. The RNA was PolyA tailed and reverse-transcribed to cDNA using the QuantiMir RT Kit (System Biosciences, CA) according to the manufacturer's instructions. pY RNA1-s2 and Y RNA1 were quantified by qRT-PCR using primers specific to the indicated small RNA and the 3′ Universal Primer provided by the QuantiMir RT Kit. The primer used for Y RNA1 is: 5′-GGCTGGTCCGAAGGTAGTGAGTTATC-3′ and the primer used for pY RNA2-s2 is 5′-CTTCTCACTACTGCACTTGACTAGTCT-3′. Several small RNAs served as controls: U5 snRNA, U6 snRNA and Sno202 RNA were quantified using t following primers: U5 snRNA, 5′-CTCTTCAGATCGTATAAATCTTTCG-3′; U6 snRNA, 5′-TGGCCCCTGCGCAAGGATG-3′ and Sno202, 5′-GACTTGATGAAAGTACTTTTGAACC-3′. Each small RNA level was calculated relative to the highest expressing RNA.

To obtain RNA from retinas from multiple species (Figure 4) a single retina from each was either divided into pieces and homogenized or in the case of the rhesus macaque, first pulverized and then dissolved in extraction buffer. The construction of cDNA libraries and qRT-PCR was performed as described above, with the exception that 500 ng RNA was used for each reaction. Expression levels are reported as copies/500 ng.

## Supporting Information

File S1 **Figures S1–S5.** Figure S1: A. Sequence analysis of Y RNA: IGV browser snapshot of Y RNA and genbank entries. Figure S2: Specificity of pY RNA1-s2 probe used for In-situ hybridization. Figure S3: Identification of Matr3 by mass spectrometry. Figure S4: Matrin 3 cytoplasmic and nuclear localization. Figure S5: Alignment of the RRM domains from Matr3.
(DOCX)
