# Supplementary material for: pY RNA1-s2: A Highly Retina-Enriched Small RNA That Selectively Binds to Matrin 3 (Matr3)
Source: PLoS One. 2014 Feb 18;9(2):e88217. doi: 10.1371/journal.pone.0088217 (PMC3928194; doi:10.1371/journal.pone.0088217)
Supplement: File S1 — Figures S1–S5. Figure S1: A. Sequence analysis of Y RNA: IGV browser snapshot of Y RNA and genbank entries. Figure S2: Specificity of pY RNA1-s2 probe used for In-situ hybridization. Figure S3: Identification of Matr3 by mass spectrometry. Figure S4: Matrin 3 cytoplasmic and nuclear localization. Figure S5: Alignment of the RRM domains from Matr3. (DOCX) [file pone.0088217.s001.docx]

**Supporting Information**

**Supplemental Figures**

**Supplemental Figure S1. A**. **Sequence analysis of Y RNA: IGV browser snapshot of Y RNA and genbank entries**.


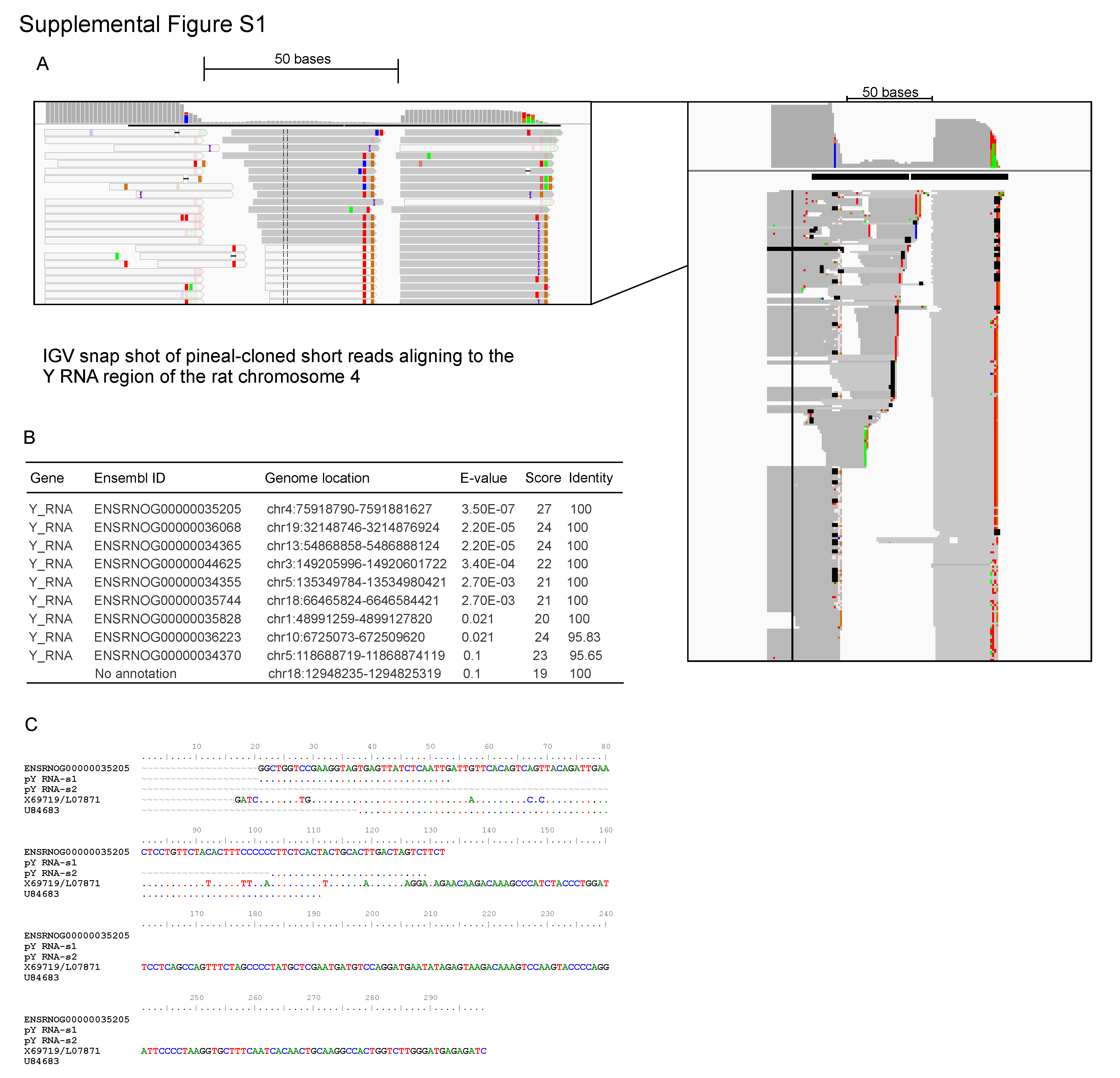


Short reads (sequenced from pineal gland) were aligned with Novoalign as described in [[17](#_ENREF_17)] and visualized in the IGV viewer [[50](#_ENREF_50)]. Outlined white boxes represent reads that map with a mapping quality of zero, indicating the read maps to other locations in the genome. Solid grey boxes indicate high quality mapping. The protocol used for the massively parallel sequencing allows for the strand to be determined and all reads aligned to the negative strand. **B**. Results of a BLAT search using pY RNA1-s2; only one location in the rat genome has an exact match with all 27 bases. Other Ensembl entries are either paralogues or homologies of Y RNA. **C**. Alignment of an incomplete nucleotide sequences present in Genbank that aligns to Y RNA1. The predicted gene ENSRNOG00000035205 is shown as the base sequence for this alignment; pY RNA1-s1 and pY RNA1-s2 align with a 100% match. X69719/L07871 align to another region of the genome (chr7:76,773,363-76,774,217) and U84683 is a partial cDNA cloned from a rat fibroblast cell line (direct submission to Genbank).

**Supplemental Figure S2. Specificity of pY RNA1-s2 probe used for In-situ hybridization.**


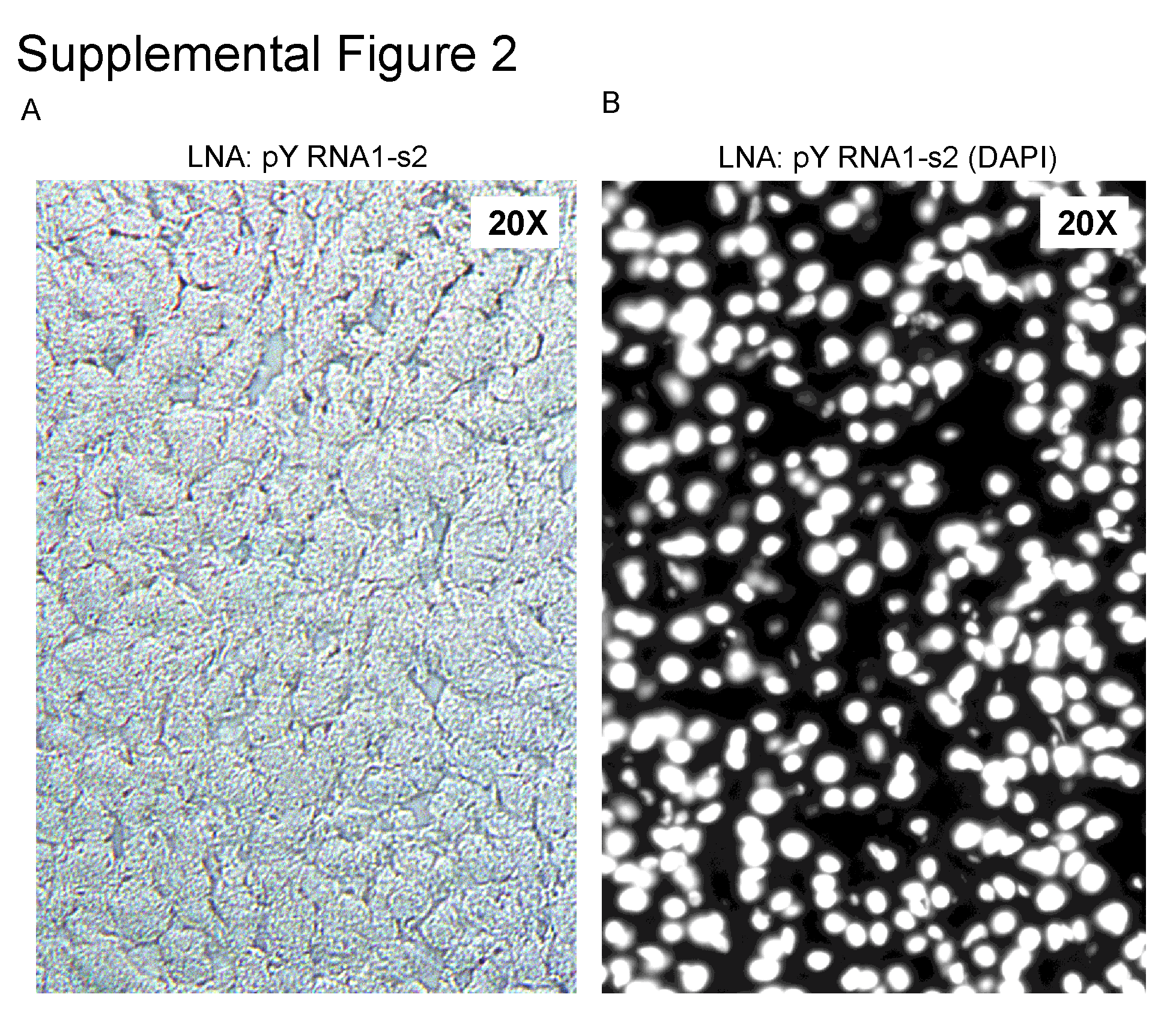
 **A.** Frozen sections (10μM) of rat liver were incubated with the same DIG-labeled probe directed against pY RNA1-s2 that was used to probe sections of retina tissue. **B**. DAPI counterstaining of the image in **A**.

**Supplemental Figure S3. Identification of Matr3 by mass spectrometry**.


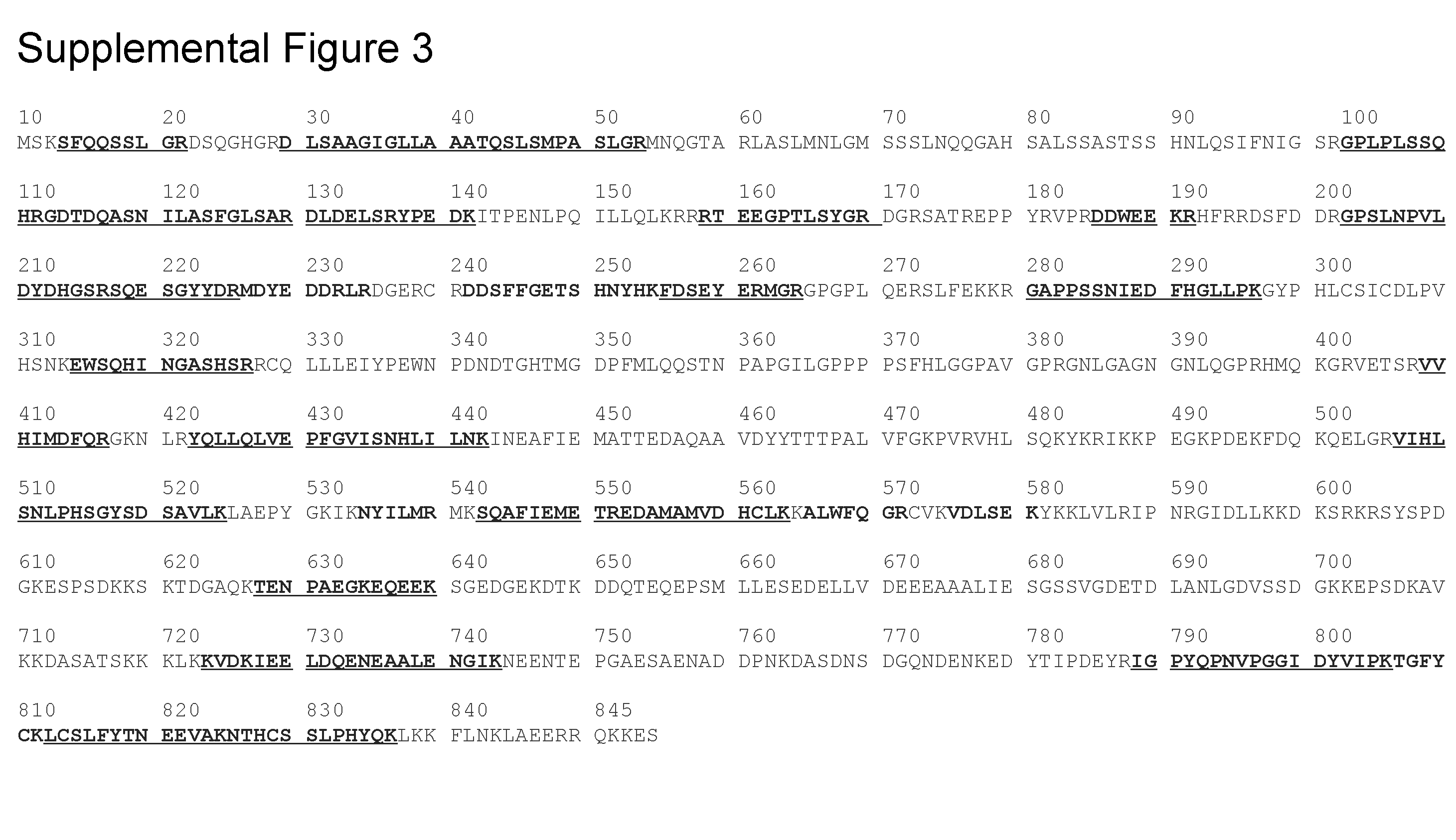


Peptides analyzed by ms/ms, from a trypsin digest of Matr3, are indicated in bold. Underlined sequences indicate peptides that were observed to undergo fragmentation to give MS/MS spectra.

**Supplemental Figure S4. Matrin 3 cytoplasmic and nuclear localization.**


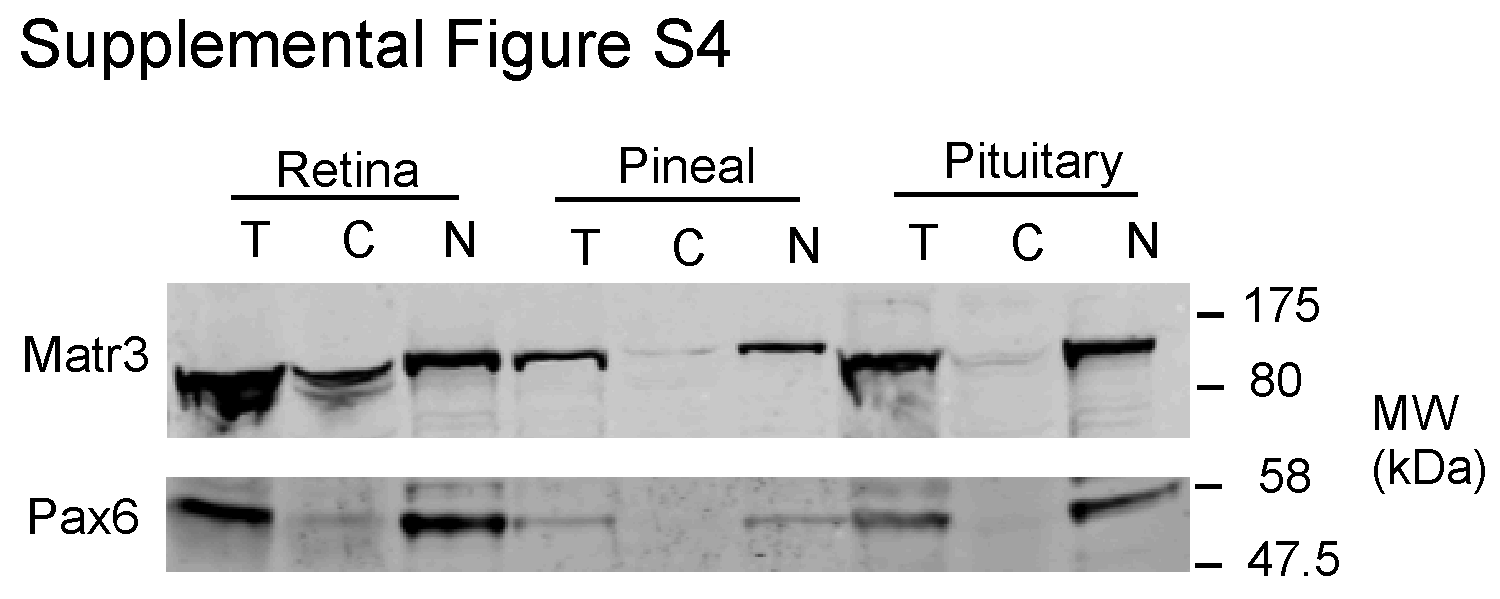
 The cytoplasmic and nuclear fractions of retina, pineal and pituitary tissues were separated as described in “Materials and Methods”, lysate prepared and equal amounts of protein subjected to SDS-PAGE followed by western blotting for Matrin 3. The subcellular fractions are indicated with: T, total lysate; C, cytoplasmic fraction and N, nuclear fraction.

**Supplemental Figure S5. Alignment of the RRM domains from Matr3.**


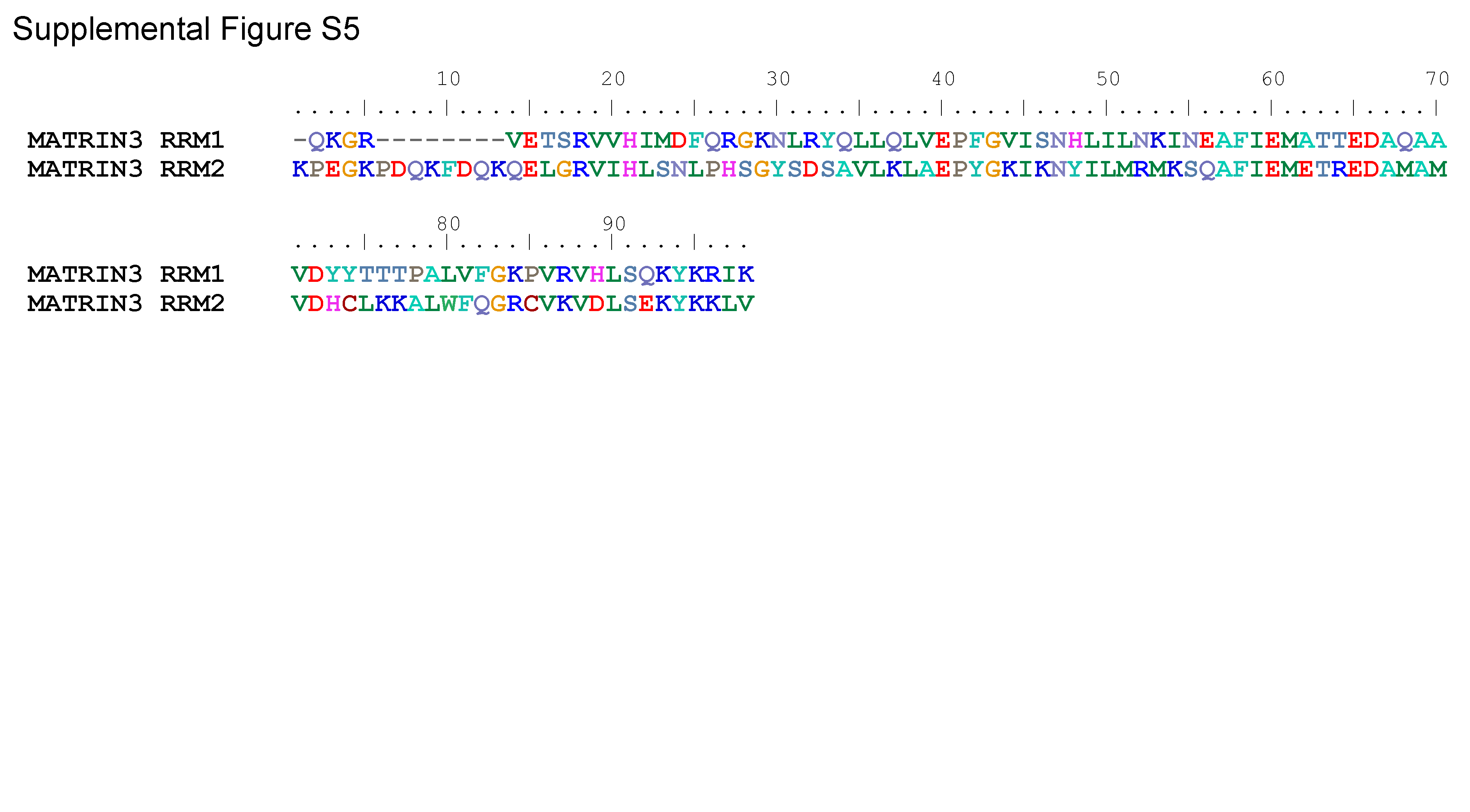


The amino acid sequence of RRM domains previously reported to bind RNA [[40](#_ENREF_40)] were aligned using the ClustalW algorithm [[51](#_ENREF_51)].
